# Applying Four-Step Characteristic Ion Filtering with HPLC-Q-Exactive MS/MS Spectrometer Approach for Rapid Compound Structures Characterization and Major Representative Components Quantification in Modified Tabusen-2 Decoction

**DOI:** 10.1155/2021/9255305

**Published:** 2021-12-31

**Authors:** Yu Zhao, Xin Dong, Zhi Wang, Rui Dong, Ren Bu, Qianxi Feng, Peifeng Xue, Bi Qu

**Affiliations:** Department of Pharmacy, Inner Mongolia Medical University, Jinshan Development Zone, Hohhot 010110, China

## Abstract

Modified Tabusen-2 decoction (MTBD) is traditional Chinese Mongolia medicine, mainly used to treat osteoporosis. However, the precise material basis of this prescription is not yet fully elucidated. Herein, we establish an HPLC-Q-Exactive MS/MS spectrometer method with four-step characteristic ion filtering (FSCIF) strategy to quickly and effectively identify the structural features of MTBD and determine the representative compounds content. The FSCIF strategy included database establishment, characteristic ions summarization, neutral loss fragments screening, and secondary mass spectrum fragment matching four steps. By using this strategy, a total of 143 compounds were unambiguously or tentatively annotated, including 5 compounds which were first reported in MTBD. Nineteen representative components were simultaneously quantified with the HPLC-Q-Exactive MS/MS spectrometer, and it is suitable for eight batches of MTBD. Methodology analysis showed that the assay method had good repeatability, accuracy, and stability. The method established above was successfully applied to assess the quality of MTBD extracts. Collectively, our findings enhance our molecular understanding of the MTBD formulation and will allow us to control its quality in a better way. At the same time, this study can promote the development and utilization of ethnic medicine.

## 1. Introduction

Tabusen-2 decoction (TBD) is composed of *Echinops latifoliu*s Tausch (ELT) and *Eucommia ulmoides* Oliver (EU) [1]. On this basis, Modified Tabusen-2 decoction (MTBD) adds *Panax notoginseng* (PN) and *Carthamus tinctorius* L. (CT) [2]. Osteoporosis is a common orthopedic disease, especially in the elderly and postmenopausal women in China. TBD is a traditional classic prescription; it has been used to treat osteoporosis for centuries [3]. The literature shows that MTBD has the effect of treating osteoporosis; it can also be used to promote blood circulation, relieve swelling, relieve pain, continue muscles and bones, and treat soft tissue contusions, crush injuries, joint sprains, trauma, and open trauma caused by surgery [4, 5]. The chemical compositions of each herb are various, having different pharmacological effects according to past reports. ELT, a traditional Chinese Mongolia herb, contained isochlorogenic acid A (ICGAA), chlorogenic acid, and other phenylpropanoids [6, 7], which are the main active components in herb. The pharmacological mitigation of ELT on osteoporosis of postmenopausal women was also reported [8]. EU is enriched with lignans and iridoids, including geniposidic acid (GPA) and pinoresinol diglucoside (PDG), having obvious antihypertensive effect [9]. In recent years, EU has attracted considerable attention because of its antiosteoporosis, antisenile dementia, antiaging, anti-inflammatory, antithrombotic, and antitumor activities [10, 11]. The flavonoids are the main active components of CT, with the efficacy of promoting blood circulation, removing blood stasis, and relieving pain [12]. Varieties of natural pigments isolated from CT, such as yellow pigments and red pigments [13], not only have pharmacological functions but also have some nutritive value. Furthermore, triterpenoid saponins are main active constituents in PN, which are widely used for promoting blood clotting, relieving swelling, and alleviating pain [14].

In accordance with traditional Chinese medicine (TCM), traditional Mongolia medicine (TMM) is characterized with multiple components and multiple targets and plays different roles in clinical therapy. This means that it is a great challenge to explain the main chemical composition of MTBD by traditional analytical methods. In particular, the presence of isomers makes its separation and analysis more difficult. In order to solve this problem, some researchers have used the methods of mass defect, relative mass defect, neutral loss filtering (NLF), mass defect filtering, and precursor ion to characterize the chemical structure in TCM or TMM prescription [15–19]. It has vital-important reference value for our following experiment. With the promotion of high-resolution mass spectrometry [20, 21], we propose an FSCIF strategy for substructure recognition, which can significantly improve the detection effectiveness, accuracy, and sensibility. This analysis program shows obvious efficiency (reduce data processing time) and intelligence (simplify the process of structural identification).

Xie et al. [22] determined hydroxysafflor yellow A, notoginsenoside R_1_, ginsenoside Rg_1_, and ginsenoside Rb_1_ with HPLC, but there are disadvantages of insufficient sensitivity and long running time (40 min). Hua et al. [23] established an HPLC-ELSD method to quantify the content of notoginsenoside R_1_, ginsenoside Rg_1_, and ginsenoside Re in PN but did not determine the content of the main components of ELT, EU, and CT. Hua et al. [24] conducted three different experiments by using HPLC, Ultraviolet detection, and ELSD methods and finally measured the content of representative components of ELT, EU, PN, and CT. But the shortcomings of this method are cumbersomeness and low responsiveness and they cannot be ignored. On the other hand, the previous literature has qualitatively analyzed the ingredients in a single medicinal material; it is not enough to explain the overall structure of MTBD due to the interaction between temperature and herbs in the process of decoction.

In order to explore the material basis of MTBD, clarify the composition of the compounds, and determine the content of the compounds, this experiment used the HPLC-Q-Exactive MS/MS spectrometer method to conduct a comprehensive material basis determination of MTBD, which provided a foundation for the subsequent quality standard formulation; it also provided guarantee for pharmacodynamic and pharmacokinetic research. Besides, 143 compounds were unambiguously or tentatively annotated with FSCIF strategy, including 5 compounds which were first reported in MTBD. Finally, we evaluated the differences in the content of 19 compounds in samples from different preparation batches, laying a foundation for subsequent quality evaluation.

## 2. Experimental

### 2.1. Materials and Reagents

A total of four batches of ELT were collected from various areas of Inner Mongolia (including Hohhot, Ordos, Xilingol, and Ulan Hot) in August 2020 (the GPS coordinates of the plant *Echinops latifolius* Tausch collection site are 41.1206962700 and 111.4084477500). Different batches of EU, CT, and PN herbs were purchased from Bozhou Pharmaceutical Co., Ltd. (Anhui, China) and GuoDa Drugstore (Hohhot, Inner Mongolia). All herbs were authenticated by Professor Bi Qu (Department of Pharmacognosy, Inner Mongolia Medical University). These specimens were preserved in the Department of General Investigation of Traditional Chinese Medicine Resources, Inner Mongolia Medical University.

**Figure 1:**
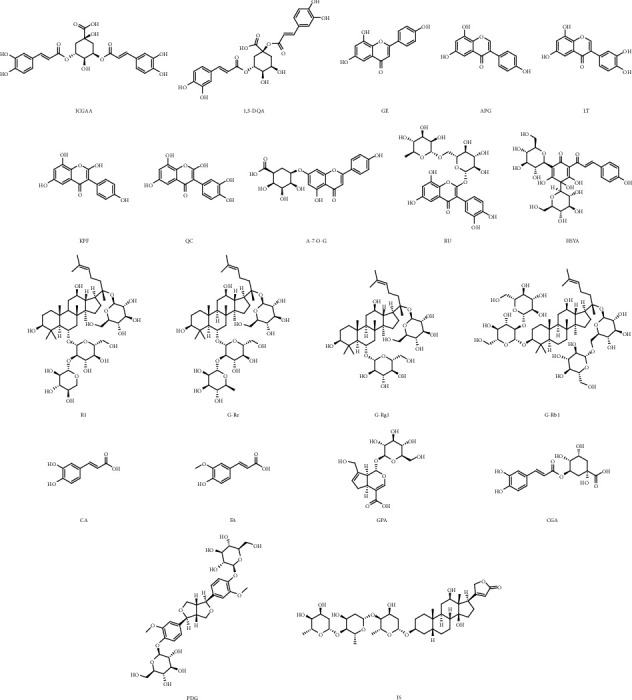
The chemical structures of nineteen analytes: isochlorogenic acid A (ICGAA), 1,5-dicaffeoylquinic acid (1,5-DQA), genistein (GE), apigenin (APG), luteolin (LT), kaempferol (KPF), quercetin (QC), apigenin-7-O-glucuronide (A-7-0-G), rutin (RU), hydroxysafflor yellow A (HSYA), notoginsenoside R_1_ (NG-R_1_), ginsenoside Re (G-Re), ginsenoside Rg_1_ (G-Rg_1_), ginsenoside Rb_1_ (G-Rb_1_), caffeic acid (CA), ferulic acid (FA), geniposidic acid (GPA), chlorogenic acid (CGA), pinoresinol diglucoside (PDG), and digoxin (internal standard, IS).

Isochlorogenic acid A (ICGAA), 1,5-dicaffeoylquinic acid (1,5-DQA), genistein (GE), apigenin (APG), luteolin (LT), kaempferol (KPF), quercetin (QC), apigenin-7-O-glucuronide (A-7-0-G), rutin (RU), hydroxysafflor yellow A (HSYA), notoginsenoside R_1_ (NG-R_1_), ginsenoside Re (G-Re), ginsenoside Rg_1_ (G-Rg_1_), ginsenoside Rb_1_ (G-Rb_1_), caffeic acid (CA), ferulic acid (FA), geniposidic acid (GPA), chlorogenic acid (CGA), pinoresinol diglucoside (PDG), and digoxin (internal standard, IS) were purchased from Cybertech Limited (Beijing, China), with HPLC purity ≥98%. The chemical structures of these 19 compounds are displayed in Figure 1. LC-MS grade methanol, acetonitrile, and formic acid were achieved from Fisher Scientific (Hampton, NH, USA). Deionized water was prepared on a Millipore water purification system (Billerica, MA, USA). The columns used in the experiment were as follows: ACE C18-PFP column (100 × 3.0 mm ID, 3 *μ*m), Grace Alltima C18 column (250 mm × 4.6 nm, 5 *μ*m), HITACHI LaChrom C18 column (250 mm × 4.6 mm ID, 5 *μ*m), and Thermo ODS-2 HYPERSIL column (250 mm × 4.6 mm, 5 *μ*m).

### 2.2. MTBD Sample and Standard Solutions Preparation

Sample preparation was a critical step for precise and convincing detection by the HPLC-Q-Exactive MS/MS spectrometer method. The MTBD samples were prepared according to our previous extraction process, and the whole operation process was in line with the basic operation safety regulations of the laboratory. EU, ELT, and CT herbal materials were powdered and sieved through 40 meshes for later extraction. A total 3.6 g of MTBD powders was accurately weighed (including 1.6 g of EU, 1.2 g of ELT, and 0.8 g of CT) and placed in a 250 mL round-bottomed flask. These powders were immersed in 50 mL ethanol: water (6 : 4, V/V) mixture and weighed and then reflux extracted twice, 90 min for each reflux. Taking into account the recovery rate of PN powder, 0.4 g PN was added before the last extraction. After merging and mixing, the solution was filtered through a 0.45 *μ*m microporous membrane. This filtrate was diluted 40 times for HPLC-Q-Exactive MS/MS spectrometer injection.

ICGAA 20.05 mg, 1,5-DQA 11.92 mg, GE 4.03 mg, APG 2.15 mg, LT 4.03 mg, KPF 1.30 mg, QC 2.02 mg, A-7-O-G 3.85 mg, RU 4.23 mg, HSYA 19.80 mg, NG-R_1_ 10.02 mg, G-Re 19.40 mg, G-Rg_1_ 10.17 mg, G-Rb_1_ 20.49 mg, CA 2.07 mg, FA 1.05 mg, GPA 4.05 mg, CGA 18.90 mg, and PDG 23.40 mg were accurately weighted and transferred into 2 mL volumetric flask, respectively. Owing to the solubility of these compounds, methanol was applied to prepare the standard solution. In order to improve the precision and accuracy of the content, digoxin was selected as the internal standard. These standard solutions were diluted with mobile phase to final concentration (Table S1) before injection into HPLC-Q-Exactive MS/MS spectrometer.

### 2.3. Chromatography and Mass Spectrometry Conditions

The characterization and quantification of MTBD sample extracts were analyzed using a Thermo HPLC-Q-Exactive MS/MS spectrometer system (HPLC, UltiMate 3000, mass system, Quadrupole Exactive Orbitrap ^TM^). The qualitative analytical conditions were as follows: HPLC column, COSMOSIL C18 (250 mm × 4.6 mm ID, 5 *μ*m); solvent system, methanol (A), and water containing 0.1% (v/v) formic acid (B); gradient program, 0–5 min, 2%–5%A; 5–10 min, 5%–10%A; 10–15 min, 10%–18%A; 15–25 min, 18%–23%A; 25–35 min, 23%–28%A; 35–55 min, 28%–33%A; 55–60 min, 33%–39%A; 60–70 min, 39%–43%A; 70–75 min, 43%–46%A; 75–85 min, 46%–60%A; 85–100 min, 60%–65%A; 100–105 min, 65%–75%A; 105–110 min, 75%–100%A; 110–130 min, 100%–100%A; flow rate, 0.6 mL/min; column temperature, 30°C; sample injection volume, 10 *μ*L. The quantitative analysis of MTBD sample extracts was separated on an ACE C18-PFP (100 × 3.0 mm ID, 3 *μ*m) column. The mobile phase consisted of methanol (A) and water containing 0.3% (v/v) formic acid (B). A gradient program was used as follows: 0–6 min, 40%–40%A; 6–15 min, 40%–90%A; 15-16 min, 90%–10%A; 16–21 min, 10%-10%A; 21-22 min, 10%–40%A; and 22–25 min, 40%-40%A. The flow rate was set as 0.3 mL/min. The column temperature was kept at 30°C. Sample injection volume was 2 *μ*L.

The qualitative and quantitative mass parameters conditions were set up as follows: auxiliary gas heater temperature, 150°C; capillary temperature, 350°C; spray voltage, 3.5 kv; S-lens RF level, 50; sheath gas flow rate, 40 L; and auxiliary gas flow rate, 2 PSI. AGC was 3 × 10^6^ in MS scan and 1 × 10^5^ in MS/MS scan; IT was 100 ms in MS scan and 50 ms in MS/MS scan; resolution was 70000 in MS scan and 17500 in MS/MS scan; NCE was set as 30 v. Scanning range was 100–1500 *m/z*. Mass spectrometry uses full scan mode for analysis in positive ion mode and negative ion mode.

### 2.4. Method Validation

The dependent variable was the ratio of the peak area of each analyte to the peak area of the internal standard, while the independent variable was set as the concentration value of each analyte; the least square regression was used to construct the standard curve equation. The intraday and interday precisions and accuracies were assessed by analyzing each concentration level (low, medium, and high) of six repeated QC samples on the same day and three consecutive days, respectively. Sample stability was investigated after the extracts were kept at room temperature for 0 h, 6 h, 12 h, and 24 h. Add the mixed control solution equal to the content of each analyte in the sample to the MTBD sample, repeat the preparation of 6 solutions, and calculate the recovery according to the following formula:

(1) $$\begin{array}{c} \text{recovery}\left(\%\right)=\frac{\left(\text{detected amount}-\text{original amount}\right)}{\text{spiked amount}}\times 100\%. \end{array}$$

## 3. Results and Discussion

### 3.1. Construction of the Identification Strategy

**Figure 2:**
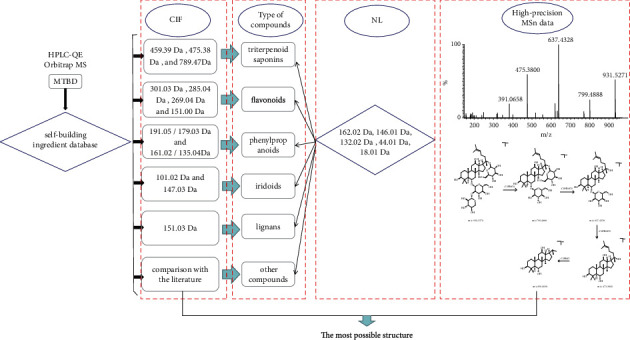
Analysis strategy of qualitative research of MTBD.

**Figure 3:**
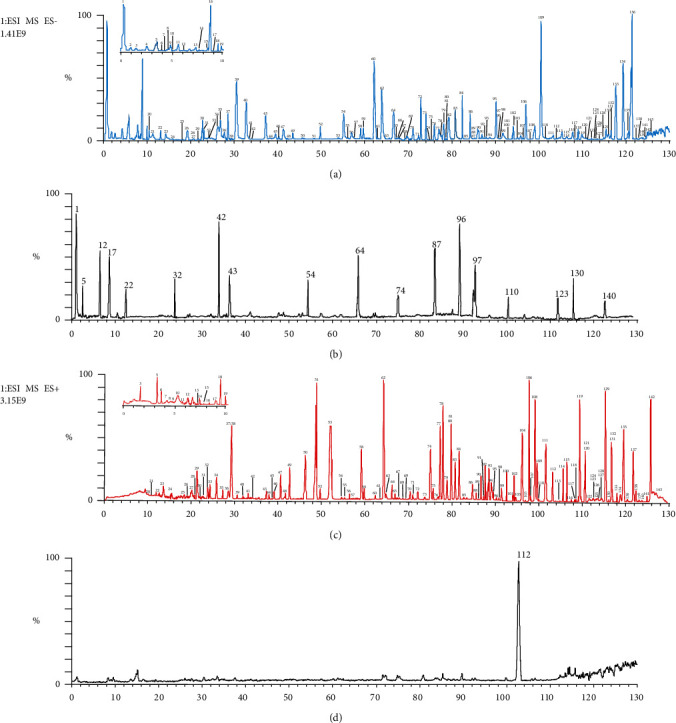
The typical total ion chromatograms (TICs) of MTBD. (a) TIC in negative ion mode. (b) Comparison with standard in negative ion mode. (c) TIC in positive ion mode. (d) Comparison with standard in positive ion mode.

Each type of compounds has its similar core and skeleton. On this basis, the characteristic ion will be produced, which provides us with new ideas for identifying these structures. In addition, FSCIF is especially suitable for compounds with the same structural type containing similar fragmentation pathways with some characteristic ions. Correspondingly, an FSCIF-based and substructure scanning strategy will be used for rapid identification of MTBD structures. The analytical strategy is shown in Figure 2. The compounds in MTBD were characterized by HPLC-Q-Exactive MS/MS spectrometer method with FSCIF strategy, including the following steps: (1) established the self-building chemical database of MTBD according to literature and online database; (2) comprehensively summarized characteristic ions for each compound type to conduct global identification of the ingredients in MTBD; (3) rapidly screened relevant structure information by neutral loss fragments (NLF) to conform the sugar type, conjunction position, and other information; (4) concluded the precise compound structure through high-precision MS/MS data. The typical total ion chromatograms (TICs) of MTBD by HPLC-Q-Exactive MS/MS spectrometer system in positive and negative ion modes are shown in Figure 3. 143 compounds were annotated through high-precision MS/MS data, including 51 triterpenoid saponins, 28 flavonoids, 20 phenylpropanoids, 15 iridoids, 12 lignans, 11 polyphenols, and 6 other types (Table 1), in which 5 compounds were first reported in MTBD and 20 compounds were unambiguously identified by comparison with reference standards. These 143 components' structures are shown in Figure S1.

### 3.2. Qualitative Analysis

#### 3.2.1. Identification of Triterpenoid Saponins

Triterpenoid saponins were typical bioactive components of PN, which were classified into two categories of protopanaxadiol (PPD) triterpenoid saponins and protopanaxatriol (PPT) triterpenoid saponins; the characteristic ions at *m/z* 459.39 [aglycones-H]^−^ and at *m/z* 475.38 [aglycones-H]^−^ corresponded to the PPD and PPT type ginsenosides [25]. In this study, most triterpenoid saponins (46 compounds) were detected [M + Na]^+^ in positive ion mode, other triterpenoid saponins (5 compounds) were detected [M − H]^−^ in negative ion mode, and excimer ion peaks can produce different cleavage modes to provide structural information such as aglycone type, sugar type, and its junction position. Compounds 110 and 123 were filtered by characteristic ion *m/z* 459.39, which tentatively identified PPD type ginsenosides; for compound 110 (C_54_H_92_O_23_) [M − H]^−^ at *m/z* 1107.5956, its molecular ion peak successively lost the four molecules of glucose and obtained *m/z* 945.5432, *m/z* 783.4906, *m/z* 621.4368, and *m/z* 459.3851. Compared with the standard, compound 110 was identified as ginsenoside Rb_1_; the possible cleavage pathways of ginsenoside Rb_1_ are shown in Figure S2-A. Similarly, [M − H]^−^ at *m/z* 945.5428 (compound 123) was tentatively identified ginsenoside Rd; the main fragment ions were [M-H-glc]^−^*m/z* 783.4907, [M-H-glc-glc]^−^*m/z* 621.4375, and [M-H-glc-glc-glc]^−^*m/z* 459.3848 [26]. Compound 87 was filtered by characteristic ion *m/z* 475.38, which tentatively identified PPT type ginsenoside. In the secondary mass spectrum, fragment ions *m/z* 799.4888, *m/z* 637.4328, *m/z* 475.3800, and *m/z* 391.0658 were [M-H-xyl]^−^, [M-H-xyl-glc]^−^, [M-H-xyl-glc-glc]^−^, and aglycon; the possible cleavage pathways of [M − H]^−^ are shown in Figure S2-B.

By using the FSCIF strategy, a total of eleven compounds (107, 112, 113, 116, 117, 118, 119, 122, 126, 127, and 129) were detected by characteristic ion of 789.47 Da ([M + Na-glcglc^6^malonyl]^+^). The retention time of compound 122 was 112.04 min; the fragment ions *m/z* 451.1044 and *m/z* 789.4733 were a pair of complementary ions [glcglc^6^malonyl + Na]^+^ and [M + Na-gicglc^6^malonyl]^+^. In addition, the fragment ions were observed in *m/z* 1173.6008, 113l.5912, 875.4739, 83l.4839, and 407.1150, which were assigned to [M + Na-CO_2_]^+^, [M + Na-malonyl]^+^, [M + Na-glcglc]^+^, [M + Na-(glcglc + CO_2_)^+^], and [glcglc^6^malonyl + Na-CO_2_]^+^ fragment ions. The possible cleavage pathways of compound 122 are shown in Figure S2-C.

Additionally, the sugar type and its junction position were concluded with the application of NLF strategy. The position of sugar fragments on the aglycon was relatively fixed (C3, C6, and C12), and the main types of sugars were glc (162.02 Da), rha (146.01 Da), and xyl (132.02 Da); the linkage between sugars is mainly l–2 and 1–6. In this experiment, nineteen compounds (62, 82, 83, 84, 89, 90, 91, 92, 93, 94, 98, 99, 102, 105, 106, 120, 124, 125, and 133) were detected by NLF with 162.02 Da. Compounds 36, 86, 103, and 104 filtered by 146.01 Da or 132.02 Da were obtained. Compound 103 (C_42_H_72_O_13_) [M + Na]^+^ at *m/z* 793.4708 tentatively annotated notoginsenoside R_2_; the main fragment ions were [M-H-xyl]^−^*m/z* 661.4249, [M-H-xyl-glc]^−^*m/z* 481.3630, and [M-H-xyl-glc-rha]^−^*m/z* 335.0939. Compound 104 (C_42_H_72_O_13_) [M + Na]^+^ at *m/z* 807.4865 tentatively annotated ginsenoside Rg_2._ First, the ion at *m/z* 661.4281 was formed by the neutral loss of a rhamnose unit of the ion at *m/z* 807.4865. Second, the ion at *m/z* 481.3676 was formed by the neutral loss of a glucose unit of the ion at *m/z* 661.4281. Finally, ion at *m/z* 349.1101 was formed by the neutral loss of a xylose unit of the ion at *m/z* 481.3676.

#### 3.2.2. Identification of Flavonoids

Most flavonoid aglycones were derivatives of quercetin, kaempferol, and apigenin, so we set 301.03 Da, 285.04 Da, and 269.04 Da as characteristic ions templates for these components annotation, which contributes to the rapid annotate flavonoids. A total of nine compounds (1, 2, 47, 51, 54, 57, 67, 80, and 81) screened with 301.03 Da were found; compound 1 showed [M − H]^−^ at *m/z* 609.1461, which was tentatively identified as rutin; its important fragment ion was 301.03 Da in secondary mass spectra, indicating the neutral loss of 308.11 Da (C_12_H_20_O_9_). In addition, the occurrences of *m/z* 283.0325, *m/z* 255.0292, and *m/z* 227.0321 were a better proof of [M-H-C_12_H_20_O_9_-H_2_O]^−^, [M-H-C_12_H_20_O_9_-H_2_O-CO]^−^, and [M-H-C_12_H_20_O_9_-H_2_O-2CO]^−^, which were the main peak, appearing in second mass spectra (Figure S3-A). Moreover, compounds 57, 80, and 81 were annotated for the first time in MTBD. Compound 57 showed [M − H]^−^ at *m/z* 771.1989; the ion at *m/z* 609.1469 was formed by the neutral loss of a glucose unit of the ion at *m/z* 771.1989. Besides, the ion at *m/z* 463.0873 was formed by the neutral loss of an xylose unit of the ion at *m/z* 609.1469. Finally, ion at *m/z* 301.0351 was formed by the neutral loss of a glucose unit of the ion at *m/z* 463.0873. Hence, compound 57 was tentatively annotated Quercetin 3-glucosyl-(1->3)-rhamnosyl-(1->6)-galactoside.

A total of five compounds (12, 71, 73, 74, and 88) acquired with 285.04 Da were found. Compound 12 showed [M − H]^−^ at *m/z* 285.0404, *m/z* 257.0453, *m/z* 239.1650, *m/z* 229.0322, and *m/z* 185.0420, corresponding to [M-H-CO]^−^, [M-H-CO-H_2_O]^−^, [M-H-2CO]^−^, and [M-H-2CO-CO_2_)^−^, which contributed to the crack of C2-C3 and C4–C10. In addition, the fracture of C4–C10 bond can also lead to the removal of C_2_H_2_O (42.02 Da), which corresponded to *m/z* 243.1601. Next, the removal of CO_2_ (44.01 Da) results in the generation of *m/z* 199.0395. By using the FSCIF strategy, seven compounds (69, 72, 85, 96, 97, 100, and 130) screened with 269.04 Da were found. Compound 97 was tentatively identified as apigenin; a high abundance secondary mass spectrometer fragment ion *m/z* 225.0555 was formed after CO_2_ (44.01 Da) loss, indicating that apigenin derivatives were easier to lose CO_2_. In addition, *m/z* 269.0455 lost one molecule, C_3_O_2_ (68.02 Da), resulting in *m/z* 201.0553. Apigenin, which is a flavonoid with double bond on the six-membered ring, can also undergo ring opening reaction of C ring, resulting in fragment ions such as *m/z* 151.0025, *m/z* 117.0328, and *m/z* 107.0124. These structural changes were also reflected at a retro-Diels-Alder (RDA) reaction [27]. Hence, the characteristic ion of RDA was set by 151.00 Da; compounds 1, 2, 41, 67, 88, 95, 96, and 97 screened with 151.00 Da were found. Compound 95 was annotated as 4,2′,3′,4′-tetrahydroxychalcone 4′-O-(2″-O-p-coumaroyl) glucoside (*m/z* 579.1507), which was being reported from MTBD for the first time. Its molecular ion peak *m/z* 269.0455 at [M − H]^−^ was observed; the fragment ions *m/z* 271.0614, *m/z* 151.0027, and *m/z* 107.0126 proved [M − H-C_15_H_16_O_7_]^−^, [M-H-C_15_H_16_O_7_-C_8_H_8_O]^−^, and [M-H-C_15_H_16_O_7_-C_8_H_8_O-C_9_H_9_O_2_]^−^.

#### 3.2.3. Identification of Phenylpropanoids

Phenylpropanoids and their derivatives, including monocaffeoylquinic acids, biscaffeoylquinic acids, and caffeoylquinic acid derivatives, were main components widely present in MTBD. Some papers [28] have previously shown that phenylpropanoids have multifaceted effects which include anti-inflammatory, antioxidant, antimicrobial, and antidiabetic activities and exhibit renoprotective, hepatoprotective, and cardioprotective effects. By using the FSCIF strategy, twenty phenylpropanoids were found; the ions at *m/z* 191.05 Da and 179.03 Da represented the base peaks of quinic acid, whereas ions at *m/z* 161.02 Da and 135.04 Da represented the base peaks of caffeic acid. Chlorogenic acid is an ester of caffeic acid and quinic acid, which indicates that chlorogenic acid contains the feature ions of both caffeic acid and quinic acid. A total of twelve compounds (4, 14, 22, 29, 30, 38, 45, 55, 56, 60, 61, and 63) were detected by *m/z* 191.05 Da and 179.03 Da. Compound 22 was tentatively identified as chlorogenic acid, producing *m/z* 191.0554, *m/z* 179.0341 (compound 32), *m/z* 173.0446, *m/z* 161.0234, *m/z* 155.0338, *m/z* 137.0322, *m/z* 135.0440, and *m/z* 93.0333, which were corresponding to [M-H-C_9_H_6_O_3_]^−^, [M-H-C_7_H_10_O_5_]^−^, [M-H-C_9_H_6_O_3_-H_2_O]^−^, [M-H-C_7_H_10_O_5_-H_2_O]^−^, [M-H-C_9_H_6_O_3_-2H_2_O]^−^, [M-H-C_9_H_6_O_3_-3H_2_O]^−^, [M-H-C_7_H_10_O_5_-CO_2_]^−^, and [M-H-C_9_H_6_O_3_-3H_2_O-CO_2_]^−^.  Figure S3-B shows the main cracking pathways of chlorogenic acid. Compounds 16, 32, 33, and 77 were filtered by *m/z* 161.02 Da and 135.04 Da, which were indicative of caffeic acid derivatives. Take compound 77 (C_11_H_12_O_4_) as an example; [M − H]^−^ at *m/z* 207.0662 and the secondary mass spectrometry were detected at *m/z* 179.0341 [M-H-CO]^−^, *m/z* 161.0234 [M-H-CO-H_2_O]^−^, and *m/z* 135.0440 [M-H-CO-CO_2_]^−^, which were tentatively identified as ethyl caffeate. Compounds 32 and 33 also have similar pyrolysis laws.

#### 3.2.4. Identification of Iridoids

The most basic core of iridoids is iridoid alcohol, containing cyclic ethers and alcoholic hydroxyl groups, which imply that the basic skeleton of iridoid glycosides contains a characteristic dihydropyran ring which is cis-connected to a cyclopentane unit structure. A total of 15 iridoids were detected [M − H]^−^ in negative ion mode. In the ESI^−^ mode, the fragment ion ^2,7^ F0^−^ ion at *m/z* 101.02 was obtained by the fragmentation of the aglycon part of the excimer ion, which was a characteristic ion to annotate the structure of the excimer ion [29, 30]. According to the literature [11], the ion at *m/z* 147.03 was the prominent ion of iridoids. Compounds 5, 8, 11, 13, and 40 were detected by characteristic ions 147.03 Da or 101.02 Da. Taking the derivation process of compound 8 as an example, the quasi-molecular ion peak of compound 8 was *m/z* 389.1089 [M − H]^−^, yielding a formula of C_16_H_22_O_11_. The [M − H]^−^ ion of *m/z* 227.0550 was the absence of glucose neutral fragment from *m/z* 389.1089. The fragment ions *m/z* of 209.0356 and 183.0655 were losing one molecule of H_2_O and one molecule of CO_2_ from [M − H]^−^ ion of *m/z* 227.0550. Then, the ion of m/z 183.0655 losses two molecules of H_2_O, convert to the fragment ions of *m/z* 165.0543 and *m/z* 147.0285. Consistently, the dehydration of fragment ion *m/z* 209.0356 leads to the production of *m/z* 191.0553; and the fragment ion *m/z* 147.0285 was decarboxylation of *m/z* 191.0553. The cleavage detail of each ion is displayed in Figure S3-C.

In addition, iridoid glycosides are usually connected to a glucose at the C1 position, so they are easy to lose neutral fragments such as 162.02 Da (glc), 44.01 Da (CO_2_), and 18.01 Da (H_2_O) [31]. A total of five compounds (7, 10, 21, 28, and 48) were filtered by 162.02 Da. Compound 7 (C_16_H_22_O_10_) showed [M − H]^−^ at *m/z* 373.1140; the fragment ions determined from MS/MS spectra were *m/z* 211.0606 [M-H-glc]^−^, *m/z* 193.0498 [M-H-glc-H_2_O]^−^, *m/z* 167.0703 [M-H-glc-CO_2_], and *m/z* 149.0598 [M-H-glc-CO_2_-H_2_O]; *m/z* 211.0940 (373.1140 Da-162.02 Da) were characteristic fragments of compound 7, which was tentatively annotated as geniposidic acid. To sum up, the iridoids were easier to lose the glucose neutral fragment ion 162.02 Da and obtain aglycon fragment ions and then the aglycon ions decarboxylated or dehydrated to become a series of fragments.

#### 3.2.5. Identification of Lignans

A large number of the bisepoxylignans and monoepoxylignans combine with glucose to form monoglycoside or diglycoside. Therefore, the majority of them could lose glycosyl and methyl neutral fragments first and then lose one or two molecular of CH_2_O and finally formed 151.03 Da. Therefore, characteristic ion fragment 151.03 Da was used to annotate lignans. Compounds 31, 44, 52, 53, 101, and 132 were detected by FSCIF with 151.03 Da. Compound 44 showed [M − H]^−^ at *m/z* 681.2400; the fragment ions *m/z* 519.5070, *m/z* 357.1346, and *m/z* 151.0390 were corresponding to [M-H-glc]^−^, [M-H-glc-glc]^−^, and [M-H-glc-glc-C_12_H_14_O_3_]^−^. Subsequently, compounds 37, 39, 138, and 139 were filtered by NLF with ions of 162.02 Da, 44.01 Da, or 18.01 Da.

#### 3.2.6. Other Compounds

A total of 11 polyphenols (3, 6, 9, 17, 18, 20, 23, 24, 25, 34, and 35) were recognized by FSNLF analysis. Because of the presence of hydroxyl and carboxyl groups, these compounds were filtered by 18.01 Da (H_2_O) and 44.01 Da (CO_2_). In addition, six other compounds were identified by comparison with the literature.

### 3.3. Quantification of 19 Major Compounds in MTBD

The 19 compounds quantified were the screening of osteoporosis targets by network pharmacology in the early stage of our laboratory, and then the representative and top ranked compounds were selected. Methodology analysis showed that the assay method of 19 compounds (including three pairs of isomers) had good repeatability and stability.

#### 3.3.1. Specificity

**Figure 4:**
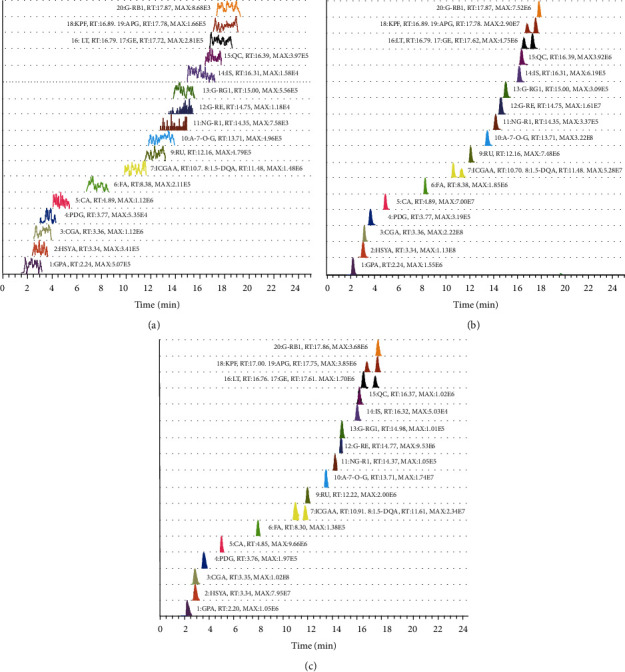
Representative chromatograms of (a) blank, (b) 19 standard samples, and (c) 19 compounds in MTBD.

The extracted ion chromatograms (EICs) of blank sample, standard mixture sample, and MTBD extracts sample are presented in Figure 4. Nineteen compounds in MTBD extracts were separated within 25 minutes, where baseline separation of each compound was achieved and no obvious signal noises occurred around determinate peak. Additionally, no interferences were detected between the three isomers.

#### 3.3.2. Linearity and Lower Limit of Quantification

Three batches of standard curve solutions with six different concentrations were prepared. The typical standard curves were assessed by using DAS 2.0 software with the quadratic weight (*W* = 1/*C*^2^). The dependent variable was the ratio of the peak area of each analyte to the peak area of the internal standard, while the independent variable was set as the concentration value of each analyte; the least square regression was used to construct the standard curve equation. The standard curves and correlation coefficients are listed in Table 2, proving the calibration curves of the components with a good linearity over the studied concentration range.

The lower limit of quantification (LLOQ) for each analyte was all with signal-to-noise ratio higher than 10, which was sufficient to perform quantitative studies of MTBD extracts.

#### 3.3.3. Precision and Accuracy

Three batches of quality control samples were prepared according to three concentration levels. Each concentration was analyzed with 6 duplications. The intraday precision values were between 1.13% and 6.66%, and the interday ones were between 2.42% and 10.62% and accuracy ranged from 86.11% to 114.27%. The above results demonstrated the acceptable precision and accuracy of the present method.

#### 3.3.4. Repeatability and Stability

Six MTBD sample extracts were prepared on the same day according to Section 2.2. The repeatability of 19 components was within 6.26% relative standard deviation (*RSD*).

Sample stability was investigated after the extracts were kept at room temperature for 0 h, 6 h, 12 h, and 24 h. The stability results of 19 compounds are summarized in Table 3; the acceptability of the data was within 3.92% deviation from the 0 h sample values, which indicated that a large number of samples could be stable in each analytical run.

### 3.4. Application to Samples Modified Tabusen-2 Decoction (MTBD)

The method established above was successfully utilized for quantitative studies of MTBD extracts, as shown in Table S2. Eight batches of MTBD samples prepared with different herb sources were determined by using the above mature method. The herb formulation of each batch is listed in Table S3. There is an indication of the fact that the concentrations of 19 compounds varied significantly in MTBD extracts; the content of flavonoids was the highest, followed by saponins (Figure S4), which attracted the attention of herb quality in picking as well as in circulating during the market. It can be seen from the quantitative research results of different batches of MTBD that we need to strictly control the quality of herb because this is the guarantee of their clinical efficacy and safety.

## 4. Discussion

Although the isolation and purification before biological activity evaluation are a traditional strategy of exploring material basis in TMM, the time-consuming and labor-intensive characteristics cannot be neglected. In quantitative experiments, Ultraviolet (UV) detector is exceedingly common for flavonoids, phenylpropanoids, and other UV-absorbing compounds [32], while it is not applicable to the analysis of saponin. Although the detection of saponin could be enabled by evaporative light scattering detector (ELSD), the sensitivity during the test procedure should also be taken into account [33]. Herein, in order to shorten the analysis time, improve the analysis sensitivity, and simultaneously determine UV-absorbing compounds and non-UV-absorbing compounds, high performance liquid chromatography coupled with mass spectrometry (HPLC-Q-Exactive MS/MS spectrometer) approach [34], as a high efficiency, is employed in this study to separate and identify the material basis in MTBD. Additionally, the existence of isomer (ICGAA with 1,5-DQA, GE with APG, LT with KPF) in MTBD increases the difficulty of separation and analysis [35]. The chromatographic conditions in quantitative analysis need to be optimized carefully during the present research.

In order to achieve better separation effect for three pairs of isomers in MTBD, the mobile phase was screened in this experiment. The peak of each component was with symmetrical shape and no tailing phenomenon. Additionally, the influence of column temperature and flow rate was considered, and a better separation was achieved under column temperature of 30°C and flow rate of 0.3 mL/min. A variety of chromatographic columns were also optimized in this study. Compared with ACE C18-PFP column (100 × 3.0 mm ID, 3 *μ*m), Grace Alltima C18 column (250 mm × 4.6 nm, 5 *μ*m), HITACHI LaChrom C18 column (250 mm × 4.6 mm ID, 5 *μ*m), and Thermo ODS-2 HYPERSIL column (250 mm × 4.6 mm, 5 *μ*m), ACE C18-PFP column had better separation and resolution, especially for the three isomers.

It was found through analysis that the contents of the 19 components differ in MTBD prepared from different batches of crude drugs; this might be because the crude drugs of different batches were different in origin, growing environments, and harvest time. This has aroused our attention in all aspects of picking and transportation. The presence of moisture will affect the determination of the content of the active ingredients in the medicinal materials. Therefore, the near-infrared method was used in the study to detect the moisture content in the relevant medicinal materials to ensure the final quantitative accuracy of the effective ingredients [36]. Refluxing was used to prepare the MTBD in the present study [37]. Furthermore, some literatures [38–42] have carried out assays on HSYA, RU, QC, G-Rb_1_, G-RG_1_, NG-R_1_, G-Re, FA, LT, KPF, APG, and GE; but the HPLC-Q-Exactive MS/MS spectrometer approach displayed distinct superiority with desirable resolution and Lower LLOQ. The previous literature [43, 44] measured the content of CA, 1.5-DQA, GPA, PDG, and CGA, but it took too long (60 minutes) and restricted its modern development. Some studies [45, 46] have shown the contents of ICGGA and A-7-O-G; on this basis, we can have a wider linear range and have greater reference value for the formulation of the content of different batches of samples.

## 5. Conclusions

Based on HPLC-Q-Exactive MS/MS spectrometer with FSCIF approach to rapid detection of structure fragment and quantification of major representative components in MTBD, 143 compounds with seven chemical categories were unambiguously or tentatively identified. This study not only enriched the cleavage law of MTBD compounds but also established an approach for the accurate search and discovery of active components from complex mixtures. The repeatability, accuracy, stability, linearity, recoveries, and reproducibility of quantitative analysis all meet the criteria for acceptability of quantitative studies. The determination of 19 compounds in MTBD extracts in different batches was obtained to monitor the quality of each prescription, which facilitates the better development of quality evaluation technique in MTBD and will help for further exploration of quality control of MTBD. The 19 compounds determined based on the qualitative and quantitative results are the major components of the MTDB. This experiment can provide a research foundation for subsequent pharmacokinetic studies and formulation of quality standards.

All in all, we compared the differences in the content of the same compound in the same herbs. Our quantitative method can determine 19 compounds in a short time (25 minutes), with a wider linear range and lower LLOQ. On the other hand, we compared the content difference of the same compound in different herbs, and the content fluctuation range is relatively large, which may be related to the processing, compatibility, and the changes in the decocting process of herbs. The content range of the 19 compounds that we have measured can provide the fluctuation range of the compound content when formulating quality standards in the future and help formulate content determination standards for preparations. This qualitative and quantitative analysis of MTBD could provide a new tool for the quality control of this preparation or its related TCM.

## Figures and Tables

**Table 1:** Characterization of chemical constituents of MTBD.

No.	tR (min)	Formula	Identification	Precursor ions (*m*/*z*)	Diff (ppm)	Fragment (*m*/*z*)	Type	Reference standard
1	0.25	C_27_H_30_O_16_	Rutin	609.1461[M − H]^−^	−3.414	301.0351, 300.0278, 283.0325, 271.0251, 255.0292, 227.0321, 151.0293	F	Yes
2	1.05	C_15_H_10_O_7_	Quercetin	301.0353[M − H]^−^	3.724	273.0405, 257.0452, 229.0500, 178.9978, 151.0026, 121.0283, 107.0126	F	
3	1.68	C_8_H_8_O_4_	Vanillic acid	167.0349 [M − H]^−^	0.867	152.0105, 123.0438, 108.0203	PO	
4	2.35	C_17_H_20_O_9_	Methyl chlorogenic acid	367.1034[M − H]^−^	5.239	191.0553, 173.0078	P	
5	3.57	C_11_H_14_O_5_	Genipin	225.0768[M − H]^−^	2.738	207.0659, 147.0441, 123.0439, 101.0231	I	Yes
6	3.82	C_6_H_6_O_3_	Pyrogallic acid	125.0244[M − H]^−^	−1.604	107.4741, 97.0282	PO	
7	4.09	C_16_H_22_O_10_	Geniposidic acid	373.1140[M − H]^−^	−6.227	211.0940, 193.0498, 167.0703, 149.0598, 123.0439	I	
8	4.47	C_16_H_22_O_11_	Deacetyl asperulosidic acid	389.1089[M − H]^−^	3.167	227.0550, 209.0356, 191.0553, 183.0655, 165.0541, 147.0285, 139.0389	I	
9	4.85	C_10_H_14_O_10_	2-Methylsuccinyl-6′-O-glucoside	293.0514[M − H]^−^	6.047	131.0450	PO	
10	4.92	C_15_H_22_O_9_	Aucubin	345.1191[M − H]^−^	2.335	183.0660, 165.0543, 139.0391, 121.0285	I	
11	5.37	C_17_H_26_O_11_	Harpagide acetate	405.1402[M − H]^−^	−1.364	191.0554, 147.0289, 119.0026, 101.0023	I	
12	6.23	C_15_H_10_O_6_	Kaempferol	285.0404[M − H]^−^	4.615	257.0453, 243.1601, 239.1650, 229.0322, 199.0395, 185.0420	F	Yes
13	7.41	C_23_H_34_O_15_	Genipin gentian diglycoside	549.1824[M − H]^−^	5.378	387.2035, 207.1128, 179.0551, 147.0298	I	
14	7.50	C_25_H_24_O_11_	3-Caffeoyl-5-coumaroyl-quinic acid	499.12458[M − H]^−^	5.81	353.1080, 191.0554	P	
15	8.32	C_19_H_18_O_11_	Isomangiferin	421.0776[M − H]^−^	−1.966	259.0224	F	
16	8.44	C_9_H_6_O_3_	Umbelliferone	161.0244[M − H]^−^	−0.249	135.0441, 99.0438, 71.0124	P	
17	8.99	C_7_H_6_O_5_	Gallic acid	169.0142[M − H]^−^	0.179	125.0232, 141.0914	PO	Yes
18	9.25	C_6_H_6_O_4_	2-Hydroxyphenol	141.0193[M − H]^−^	3.247	123.0175	PO	
19	9.76	C_15_H_14_O_6_	L-Epicatechin	289.0717[M − H]^−^	7.333	271.0235, 245.0411, 205.2713, 179.0110	F	
20	10.56	C_4_H_4_O_4_	Maleic acid	115.0036[M − H]^−^	−0.479	71.0124	PO	
21	11.43	C_15_H_24_O_10_	Harpagide	363.1296[M − H]^−^	0.977	183.0652, 89.0228	I	
22	12.53	C_16_H_18_O_9_	Chlorogenic acid	353.0878[M − H]^−^	0.854	191.0554, 179.0341, 173.0446, 161.0234, 155.0338, 137.0322, 135.0440, 93.0333	P	Yes
23	14.13	C_8_H_8_O_4_	Methyl protocatechuic acid	167.0349[M − H]^−^	0.508	152.0106, 123.0439, 108.0203	PO	
24	15.84	C_8_H_8_O_4_	Isovanillic acid	167.0349[M − H]^−^	−2.536	123.0439	PO	
25	18.23	C_13_H_16_O_9_	Protocatechuic acid-4-glucoside	315.0721[M − H]^−^	−1.392	108.0204	PO	
26	19.56	C_14_H_18_O_9_	4-Glucopyranoxy-3-benzoic acid	329.0878[M − H]^−^	4.340	167.0340, 152.0105, 123.0439, 108.0204	O	
27	20.23	C_9_H_12_O_5_	Rehmaglutin C	199.0611[M − H]^−^	3.316	155.0704, 137.0596	I	
28	20.39	C_18_H_24_O_12_	Asperulosidic acid	431.1194[M − H]^−^	−3.114	269.0198, 251.0098	I	
29	21.77	C_16_H_18_O_9_	Neochlorogenic acid	353.0878[M − H]^−^	−0.699	191.0554, 179.0341, 135.0440	P	
30	22.36	C_16_H_18_O_9_	4-Caffeoylquinic acid	353.0878[M − H]^−^	3.941	191.0554, 179.0340, 135.1440	P	
31	23.28	C_20_H_24_O_7_	Cycloolivil	375.1449[M − H]^−^	−2.075	327.1343, 297.1207, 257.1132, 151.0752	L	
32	24.28	C_9_H_8_O_4_	Caffeic acid	179.0349[M − H]^−^	0.856	135.0440	P	Yes
33	24.36	C_9_H_10_O_4_	Dihydrocaffeic acid	181.0506[M − H]^−^	1.849	163.0390, 135.0441, 119.0488	P	
34	26.50	C_7_H_6_O_4_	Gentianic acid	153.0193[M − H]^−^	−0.295	109.0282	PO	
35	27.23	C_7_H_6_O_4_	Protocatechuic acid	153.0193[M − H]^−^	0.685	109.0283, 91.0175,	PO	
36	28.03	C_42_H_70_O_12_	Ginsenoside F_4_	789.4759[M + Na]^+^	−1.837	707.1499, 643.4222, 349.1090	T	
37	28.55	C_20_H_24_O_7_	Oleoresin	375.1449[M − H]^−^	−1.018	179.0341, 161.0233	L	
38	29.40	C_16_H_18_O_9_	Cryptochlorogenic acid	353.0878[M − H]^−^	3.516	191.0554, 179.0340, 173.0446, 135.0440	P	
39	30.90	C_20_H_24_0_7_	Olivil	375.1449[M − H]^−^	−2.635	327.1360, 195.1251, 179.0341, 161.0220	L	
40	32.26	C_17_H_24_O_10_	Geniposide	387.1296[M − H]^−^	3.128	207.1025, 123.0444, 101.0232	I	
41	33.49	C_33_H_44_O_19_	Naringin dihydrochalcone 4-O-*β*-D-glucoside	743.2404[M − H]^−^	3.128	373.1295, 313.1088, 181.0498, 151.0396	F	
42	34.26	C_27_H_32_O_16_	Hydroxysafflor yellow A	611.1617[M − H]^−^	2.207	491.1200, 473.1092, 403.1042, 325.0720	F	Yes
43	37.58	C_10_H_10_O_4_	Ferulic acid	193.0506[M − H]^−^	2.208	178.0264, 149.0598, 134.0362	P	Yes
44	38.11	C_32_H_42_O_16_	Pinoresinol diglucoside	681.2400[M − H]^−^	2.039	519.5070, 357.1346, 151.0390, 136.0159	L	
45	39.59	C_16_H_18_O_8_	3-O-p-Coumaroylquinic acid	337.0928[M − H]^−^	6.688	191.0553, 173.0448, 163.0390	P	
46	40.21	C_27_H_36_O_13_	Citrusin B	567.2083[M − H]^−^	−4.289	341.1384, 329.1394	L	
47	41.77	C_27_H_30_O_17_	Quercetin-3, 4′-O-di-*β*-glucopyranoside	625.1410[M − H]^−^	0.831	463.0884, 301.0350, 271.0243	F	
48	42.23	C_15_H_26_O_9_	Eucommioside	349.1504[M − H]^−^	−1.102	187.1528, 89.0230	I	
49	43.69	C_10_H_10_O_3_	Coniferyl aldehyde	177.0557[M − H]^−^	−0.101	162.0312	P	
50	46.23	C_20_H_22_O_7_	Erythroglycerin-*β*-terpineol aldehyde ether	373.1292[M − H]^−^	4.259	177.0548, 165.0547, 150.0308,	P	
51	48.38	C_26_H_28_O_16_	Quercetin 3-O-sambubioside	595.1305[M + H]^+^	−0.638	301.0327	F	
52	49.62	C_26_H_32_O_11_	Pinoresinol-4′-O-*β*-D-glucopyranoside	519.1871[M − H]^−^	−3.929	357.1345, 151.0390	L	
53	54.63	C_26_H_32_O_11_	Pinoresinol-*β*-D-glucoside	519.1871[M − H]^−^	3.334	357.1345, 342.1107, 311.1293, 151.0390, 136.0154	L	
54	55.16	C_21_H_20_O_12_	Isoquercitrin	463.0881[M − H]^−^	3.623	301.0349, 271.0321, 255.0299	F	Yes
55	56.44	C_22_H_28_O_14_	5-(3 ′-o-caffeoylglucosyl) quinine	515.1406[M − H]^−^	8.420	191.0555, 161.0234, 135.0440	P	
56	57.45	C_22_H_28_O_14_	1-O-(3 ′-o-caffeoylglucosyl) quinine	515.1406[M − H]^−^	2.499	179.0341, 173.0446, 161.0233, 135.0440	P	
57^*∗*^	57.73	C_33_H_40_O_21_	Quercetin 3-glucosyl-(1->3)-rhamnosyl-(1->6)-galactoside	771.1989[M − H]^−^	2.070	609.1469, 463.0873, 301.0351	F	
58	58.66	C_28_H_36_O_13_	Syringaresionl-O-*β*-D-g1ucopyranoside	579.2083[M − H]^−^	3.298	417.1557	P	
59	59.50	C_35_H_60_O_6_	Daucosterol	575.4317[M − H]^−^	2.329	397.7564	T	
60	62.59	C_25_H_24_O_12_	1,5-Dicaffeoylquinic acid	515.1194[M − H]^−^	2.499	353.0881, 191.0554, 135.0440	P	
61	63.34	C_25_H_24_O_12_	Isochlorogenic acid A	515.1194[M − H]^−^	1.288	353.0881, 191.0554, 179.0341, 173.0446, 135.0440	P	
62	64.00	C_42_H_72_O_15_	6-O-*β*-D-Glucopyranosyl-20-o-*β*-D-glucopyranosyl-3*β*,6*β*,12*β*,20 (S)7-25-pentaphydroxydammar-23-enedroginsenoside Rg_1_	839.4763[M + Na]^+^	−2.644	659.4114	T	
63	65.10	C_25_H_24_O_12_	Isochlorogenic acid B	515.1194[M − H]^−^	1.288	353.0881, 335.0777, 191.0554, 179.0341, 173.0446	P	
64	66.70	C_9_H_16_O_4_	Eucommitol	187.0975[M − H]^−^	1.521	169.0861, 143.1068, 125.0960	I	Yes
65	66.72	C_6_H_4_O_4_	Coumalic acid	139.0036[M − H]^−^	6.332	119.5097	O	
66	67.26	C_18_H_16_O_5_	Sideroxylin	311.0924[M − H]^−^	2.177	267.0663	F	
67	67.68	C_21_H_20_O_12_	Hyperoside	463.0882[M − H]^−^	4.206	301.03455, 151.00258	F	
68	68.78	C_15_H_26_O_7_	2-(5-Hydroxyethyl-2,3-dimethyl-2-cyclopenten-1-yl)-glucopyranoside	317.1605[M − H]^−^	4.580	243.1238, 225.1132	I	
69	69.40	C_21_H_20_O_10_	Apigenin-7-O-glucuronide	431.0983[M − H]^−^	3.147	269.0376	F	
70	70.29	C_9_H_16_O_3_	1-Deoxyeucommitol	171.1026[M − H]^−^	0.930	127.1118, 125.0959	I	
71	72.63	C_21_H_20_O_11_	Astragalin	447.0933[M − H]^−^	2.532	285.0395, 241.0829, 217.0886	F	
72	73.55	C_21_H_18_O_11_	Baicalin	445.0776[M − H]^−^	4.724	269.0456	F	
73	74.23	C_27_H_30_O_15_	Nicotiflorin	593.1511[M − H]^−^	3.681	285.0404, 255.0307, 227.0352	F	
74	74.88	C_27_H_30_H_15_	Safflor yellow (A)	593.1511[M − H]^−^	3.884	285.0404	F	Yes
75	75.61	C_18_H_14_O_6_	Milletenin C	325.0717[M − H]^−^	2.650	310.0848	F	
76	76.26	C_12_H_16_O_3_	3-Butyl-4-hydroxy-4,5-dihydro-2-benzofuran-1(3H)-one	207.1026[M − H]^−^	0.368	135.0443	O	
77	77.03	C_11_H_12_O_4_	Ethyl caffeate	207.0662[M − H]^−^	3.162	179.0341, 161.0234, 135.0440	P	
78	77.96	C_48_H_82_O_19_	Notoginsenoside R_6_	985.5342[M + Na]^+^	−2.049	365.1045, 305.0816	T	
79	78.56	C_48_H_82_O_19_	Notoginsenoside R_3_	985.5342[M + Na]^+^	2.402	645.4159, 365.1044	T	
80^*∗*^	79.20	C_28_H_32_O_16_	6-Methoxykaempferol 3-robinobioside	623.16175[M − H]^−^	1.524	315.0509, 301.0320, 300.0276	F	
81^*∗*^	79.40	C_29_H_36_O_15_	3,4,6-Trihydroxy-4,2′-dimethoxychalcone 4′-O-rutinoside	623.19814[M − H]^−^	6.18	315.0510, 301.0313, 300.0376	F	
82	79.58	C_48_H_82_O_19_	Notoginsenoside M	985.5342[M + Na]^+^	−3.227	805.4688, 365.1047	T	
83	81.76	C_48_H_82_O_19_	Notoginsenoside N	985.5342[M + Na]^+^	−2.983	805.4689	T	
84	82.77	C_48_H_82_O_19_	20-O-Glucoginsenoside Rf	985.5342[M + Na]^+^	−1.684	805.4689, 365.2320	T	
85^*∗*^	83.83	C_23_H_22_O_11_	Apigenin 7-(2″-acetylglucoside)	473.1089[M − H]^−^	1.569	413.0891, 269.0379	F	
86	84.34	C_41_H_68_O_12_	Notoginsenoside T_5_	775.4602[M + Na]^+^	−2.385	692.0035, 643.3312, 463.3556, 335.0930	T	
87	84.47	C_47_H_80_O_18_	Notoginsenoside R_1_	931.5271[M − H]^−^	0.633	799.4888, 637.4328, 475.3800, 391.0658	T	Yes
88	85.37	C_15_H_10_O_6_	Luteolin	285.0404[M − H]^−^	4.720	257.0453, 151.0030	F	
89	85.76	C_42_H_72_O_14_	Majoroside F_4_	823.4814[M + Na]^+^	−1.603	643.4166	T	
90	86.17	C_42_H_72_O_14_	3-O-*β*-D-Glucopyranosyl-6-O-*β*-D-glucopyranosyl-20-(S)-protopanaxatriol	823.4814[M + Na]^+^	−3.157	703.0069, 643.4163	T	
91	87.18	C_42_H_72_O_14_	Gynoside B	823.4814[M + Na]^+^	−1.603	643.4164	T	
92	87.69	C_42_H_72_O_14_	Ginsenoside Rg_1_	823.4814[M + Na]^+^	−1.603	643.4104	T	
93	88.21	C_45_H_74_O_17_	Malonyl ginsenoside Rg_1_	909.4818[M + Na]^+^	−1.891	865.4895, 729.4166, 685.4270	T	
94	89.59	C_48_H_80_O_19_	Notoginsenoside G	983.5186[M + Na]^+^	−2.897	803.4535	T	
95^*∗*^	89.94	C_30_H_28_O_12_	4,2′,3′,4′-Tetrahydroxychalcone 4′-O-(2″-O-p-coumaroyl) glucoside	579.1507[M − H]^−^	0.622	271.0614, 151.0027, 107.0126	F	
96	90.05	C_15_H_10_O_5_	Genistein	269.0455[M − H]^−^	4.572	225.0554, 201.0555, 151.0027, 117.0329, 107.0124	F	Yes
97	90.57	C_15_H_10_O_5_	Apigenin	269.0455[M − H]^−^	4.572	225.0555, 201.0553, 151.0025, 117.0328, 107.0124	F	Yes
98	91.24	C_41_H_70_O_13_	Pseudoginsenoside RT_3_	793.4708[M + Na]^+^	3.882	613.4072	T	
99	91.79	C_44_H_74_O_15_	Yesanchinoside D	865.4919[M + Na]^+^	−4.633	685.4267	T	
100	92.47	C_30_H_26_O_12_	Apigenin-7-O-(6″-coumaroyl) glucoside	577.1351[M − H]^−^	1.390	431.0988, 269.0457	F	
101	92.96	C_20_H_22_O_6_	Epipinoresinol	357.1343[M − H]^−^	1.216	151.1533, 136.0809, 121.0282	L	
102	94.52	C_42_H_72_O_14_	Ginsenoside Rf	823.4814[M + Na]^+^	−2.113	661.5368, 641.4468, 365.1043	T	
103	95.39	C_41_H_70_O_13_	Notoginsenoside R_2_	793.4708[M + Na]^+^	−2.583	661.4249, 481.3630, 335.0939	T	
104	95.89	C_42_H_72_O_13_	Ginsenoside Rg_2_	807.4865[M + Na]^+^	−1.362	661.4281, 481.3676, 349.1101	T	
105	96.47	C_36_H_62_O_9_	Gypenoside LXXVI	661.4286[M + Na]^+^	2.479	601.2890, 481.3620	T	
106	97.28	C_36_H_62_O_9_	Ginsenoside Rh_1_	661.4286[M + Na]^+^	−1.769	481.3650, 413.2539	T	
107	98.42	C_59_H_100_O_27_	Ginsenoside Ra_3_	1263.6344[M + Na]^+^	−2.327	789.4784, 497.1457, 437.1239	T	
108	99.42	C_59_H_100_O_27_	Notoginsenoside Fa	1263.6344[M + Na]^+^	−0.688	921.5158	T	
109	100.51	C_54_H_92_O_22_	Notoginsenoside I	1115.5972[M + Na]^+^	−1.470	773.4795, 365.1046	T	
110	101.91	C_54_H_92_O_23_	Ginsenoside Rb_1_	1107.5956[M − H]^−^	0.589	945.5432, 783.4906, 621.4368, 459.3851	T	Yes
111	102.94	C_42_H_72_O_13_	Ginsenoside Rg_3_	807.4865[M + Na]^+^	−0.904	365.1046	T	
112	103.85	C_48_H_82_O_18_	Ginsenoside Re	969.5393[M + Na]^+^	−1.908	789.4742	T	Yes
113	105.07	C_54_H_92_O_23_	Yesanchinoside E	1131.5921[M + Na]^+^	−5.768	789.4737, 365.1045	T	
114	106.08	C_38_H_64_O_10_	6′-O-Acetylginsenoside F_1_	703.4391[M + Na]^+^	0.071	481.3647	T	
115	106.58	C_56_H_94_O_24_	Quinquenoside R_1_	1173.6027[M + Na]^+^	−2.548	831.4845, 365.1044	T	
116	107.65	C_56_H_94_O_24_	6‴-O-Acetylginsenoside Rb_1_	1173.6027[M + Na]^+^	−2.326	831.4845, 789.4744, 407.1151, 347.0945	T	
117	108.75	C_53_H_90_O_22_	Ginsenoside Rb_2_	1101.5815[M + Na]^+^	−1.479	789.4740, 335.0939	T	
118	109.76	C_53_H_90_O_22_	Notoginsenoside L	1101.5815[M + Na]^+^	−1.479	789.4740	T	
119	110.33	C_57_H_94_O_26_	Malonyl ginsenoside Rb_1_	1217.5925[M + Na]^+^	−2.727	1173.5993, 875.4738, 831.4844, 789.4738	T	
120	111.31	C_48_H_82_O_17_	Vina-ginsenoside R_3_	953.5444[M + Na]^+^	−2.349	773.4788	T	
121	111.47	C_48_H_82_O_18_	Gypenoside XVII	969.5393[M + Na]^+^	−0.908	365.1048	T	
122	112.04	C_57_H_94_O_26_	3-(*β*-D-Glucopyranosyl-*β*-D-glucopyranosyl)-20-O-(6-O-malonyl-*β*-D-glucopyranosyl-*β*-D-glucopyranosyl)-3*β*,12*β*,20(S)-trihydroxydammar-24-ene	1217.5925[M + Na]^+^	−1.07	1173.6008, 1131.5912, 875.4739, 831.4839, 789.4733, 451.1044, 407.1150	T	
123	112.53	C_48_H_82_O_18_	Ginsenoside Rd	945.5428[M − H]^−^	0.857	783.4907, 621.4375, 459.3848, 375.3146	T	Yes
124	112.57	C_36_H_62_O_8_	Notoginsenoside R_7_	645.4336[M + Na]^+^	−3.249	627.3813, 465.3691	T	
125	113.48	C_36_H_60_O_8_ C_36_H_60_O_7_	Ginsenoside Rh_3_	643.4180[M + Na]^+^	−3.232	583.3644, 463.3514	T	
126	113.89	C_51_H_84_O_21_	Malonyl ginsenoside Rd	1055.5397[M + Na]^+^	3.598	875.4738, 789.4740	T	
127	114.07	C_48_H_82_O_18_	Gypenoside LXXII	969.5393[M + Na]^+^	−1.691	789.4739	T	
128	114.88	C_36_H_62_O_11_	Notoginsenoside T_4_	693.4184[M + Na]^+^	−3.763	633.3707	T	
129	115.36	C_47_H_80_O_17_	3-O-[*β*-D-Glucopyranosyl(1–2)-*β*-D-glucopyranosyl]-20-O-*β*-D-xylopyranosyl-3*β*,12*β*,20(s)-trihydroxydammar-24-ene	939.5287[M + Na]^+^	−0.872	789.4735	T	
130	116.22	C_15_H_10_O_5_	Baicalein	269.0455[M − H]^−^	4.238	197.1905	F	Yes
131	117.02	C_47_H_80_O_18_	6-O-[Xylopyranosyl-*β*-D-glucopyranosyl]-3*β*,6*β*,12*β*,20(s),25-pentahydroxydammar	811.4814[M + Na]^+^	1.208	793.3365, 751.2600, 679.2239, 499.1350, 412.1227, 335.0018	T	
132	117.70	C_20_H_22_O_6_	Pinoresinol	357.1343[M − H]^−^	1.340	313.1811, 151.1520, 136.0819	L	
133	118.87	C_42_H_72_O_13_	Ginsenoside F_2_	807.4865[M + Na]^+^	0.458	627.4217	T	
134	120.08	C_42_H_72_O_13_	Gypenoside LXXV	807.4865[M + Na]^+^	7.789	365.1045	T	
135	121.53	C_29_H_42_O_5_	Ulmoidol	469.2959[M − H]^−^	3.715	423.2238	T	
136	121.65	C_28_H_34_O_4_	Unknown	433.2384[M − H]^−^	−0.685	433.2577	O	
137	122.04	C_36_H_60_O_9_	Ginsenoside Rh_7_	659.4129[M + Na]^+^	−0.349	599.3925	T	
138	122.50	C_32_H_42_O_17_	1-Hydroxypinoresinol-4,4″-di-O-*β*-D-glucopyranoside	697.2349[M − H]^−^	0.112	535.1532, 373.0323	L	
139	122.99	C_20_H_24_O_8_	Threo-dihydroxydehy-drodiconiferyl alcohol	391.1398[M − H]^−^	−3.313	313.1747, 295.0882	L	
140	123.91	C_16_H_32_O_2_	Palmitic acid	255.2329[M − H]^−^	1.978	241.3251	O	Yes
141	124.01	C_20_H_24_O_8_	Erytho-dihydroxydehydrodiconiferyl alcohol	391.1398[M − H]^−^	−2.359	341.1587, 313.0930, 207.0832	L	
142	125.83	C_18_H_36_O_2_	Palmitic acid ethyl ester	283.2642[M − H]^−^	3.859	89.0229	O	
143	127.10	C_9_H_12_O_4_	Eucommidiol	183.0662[M − H]^−^	0.268	139.1124, 93.7235	I	

**Table 2:** Calibration curves, linear range, *r*^2^, and LOQs of 19 compounds in MTBD.

Compound	Calibration curves	Linear range (*μ*g/mL)	*r* ^2^	LLOQ (*μ*g/mL)
ICGAA	*y* = 647.937*x* + 1.476	4.800–192.000	0.9994	4.800
1,5-DQA	*y* = 364.018*x* − 0.810	6.100–244.000	0.9991	6.810
GE	*y* = 22386.297*x* − 0.191	0.023–0.920	0.9982	0.025
APG	*y* = 29960.140*x* − 0.562	0.065–2.600	0.9989	0.073
LT	*y* = 15454.517*x* − 0.090	0.015–0.600	0.9986	0.015
KPF	*y* = 9208.139*x* − 0.051	0.010–0.400	0.9983	0.010
QC	*y* = 8437.051*x* − 0.062	0.011–0.440	0.9989	0.011
A-7-O-G	*y* = 6901.196*x* + 11.968	4.200–168.000	0.9962	4.200
RU	*y* = 2560.803*x* − 0.093	0.230–9.200	0.9992	0.250
HSYA	*y* = 1104.734*x* − 0.935	9.830–392.000	0.9984	10.930
NG-R_1_	*y* = 8.300*x* − 0.016	2.400–96.000	0.9964	2.400
G-Re	*y* = 730.821*x* + 0.137	1.010–40.400	0.9963	1.010
G-Rg_1_	*y* = 21.700*x* + 0.029	6.500–260.000	0.9983	6.700
G-Rb_1_	*y* = 93.320*x* + 0.128	5.660–226.400	0.9979	5.830
CA	*y* = 183840.263*x* − 8.999	0.260–10.400	0.9985	0.260
FA	*y* = 2322.874*x* − 0.525	0.390–15.600	0.9992	0.410
GPA	*y* = 119.544*x* − 0.011	0.800–32.000	0.9987	0.800
CGA	*y* = 3414.355*x* − 5.245	5.500–220.000	0.9978	5.660
PDG	*y* = 10.556*x* − 0.018	2.180–87.200	0.9987	2.180

**Table 3:** Precision, repeatability, stability, and accuracy of 19 compounds in MTBD.

Compound	Interday precision (RSD, *n* = 3)			Intraday precision (RSD, *n* = 3)			Repeatability	Stability	Accuracy (*n* = 6, %)	
	Low	Middle	High	Low	Middle	High	(RSD, *n* = 6, %)	(RSD, *n* = 4, %)	Recovery	RSD
ICGAA	4.74	5.61	6.45	1.24	2.34	3.39	2.62	3.13	107.36	1.76
1,5-DQA	3.37	4.31	5.18	1.13	0.59	1.63	6.26	3.48	106.46	2.11
GE	2.42	7.24	4.87	2.29	2.79	0.90	2.30	2.68	106.41	2.06
APG	3.34	5.34	3.64	3.22	2.58	2.82	1.70	2.18	106.73	2.23
LT	4.05	7.84	4.53	3.13	2.81	1.09	4.75	3.92	98.21	3.01
KPF	9.47	9.00	7.98	2.18	1.54	5.51	4.94	3.60	103.12	2.37
QC	8.67	6.94	5.52	6.66	2.25	1.35	4.68	2.81	103.42	4.10
A-7-O-G	4.17	3.42	7.38	4.15	3.05	3.13	2.09	2.38	104.56	1.87
RU	4.03	3.23	3.42	1.86	1.92	2.57	2.99	2.34	105.39	0.92
HSYA	3.81	4.97	5.67	2.25	1.13	4.78	3.40	2.56	104.80	1.13
NG-R1	8.22	4.19	5.85	1.63	2.07	4.55	4.05	0.75	104.87	2.81
G-Re	6.50	3.92	5.41	3.17	1.38	2.47	4.02	3.23	107.35	1.73
G-Rg_1_	6.10	7.59	6.12	5.08	4.64	4.80	3.27	2.26	104.64	1.59
G-Rb_1_	10.62	3.37	3.61	2.94	2.71	3.47	4.50	2.99	106.94	2.54
CA	6.29	5.83	4.08	5.45	3.62	4.31	2.15	2.43	101.10	0.53
FA	8.76	7.21	7.61	3.90	4.15	2.40	2.94	3.74	105.71	2.10
GPA	8.36	6.89	10.52	2.64	0.54	4.11	3.82	3.59	107.60	3.01
CGA	2.74	5.12	2.82	2.52	1.25	3.50	2.99	2.24	106.59	2.11
PDG	6.95	7.32	7.71	2.69	2.78	4.11	2.67	1.97	92.08	4.57

## Data Availability

The methodological data and structural data used to support the findings of this study are included within the article. The cleavage pathways data used to support the findings of this study are included within the Supplementary Materials.

## Supplementary Materials

Supplementary Materials Table S1: the final concentration of 19 standard solutions. Table S2: the content of 19 compounds in the MTBD medicinal measure (*μ*g, *X* ± SD, *n* = 3). Table S3: different sources of medicinal materials. Table S4: quality control concentration levels of 19 compounds. Figure S1: structures of 143 compounds. (A) Structure of flavonoids. (B) Structure of phenylpropanoids. (C) Structure of lignans. (D) Structure of iridoids. (E) Structure of polyphenol. (F) Structure of saponins. (G) Structure of other type compounds. Figure S2: the cracking pathways of (A) ginsenoside Rb_1_, (B) notoginsenoside R_1_, and (C) 3-(*β*-D-glucopyranosyl-*β*-D-glucopyranosyl)-20-O-(6-O-malonyl-*β*-D-glucopyranosyl-*β*-D-glucopyranosyl)-3*β*,12*β*, 20(S)-trihydroxydammar-24-ene. Figure S3: the cracking pathways of (A) rutin, (B) chlorogenic acids, (C) deacetyl asperulosidic acid. Figure S4: the content of eight batches of medicinal materials.
