# Supplementary material for: Applying Four-Step Characteristic Ion Filtering with HPLC-Q-Exactive MS/MS Spectrometer Approach for Rapid Compound Structures Characterization and Major Representative Components Quantification in Modified Tabusen-2 Decoction
Source: Evid Based Complement Alternat Med. 2021 Dec 31;2021:9255305. doi: 10.1155/2021/9255305 (PMC8741372; doi:10.1155/2021/9255305)
Supplement: Supplementary Materials — Table S1: the final concentration of 19 standard solutions. Table S2: the content of 19 compounds in the MTBD medicinal measure (μg, X ± SD, n = 3). Table S3: different sources of medicinal materials. Table S4: quality control concentration levels of 19 compounds. Figure S1: structures of 143 compounds. (A) Structure of flavonoids. (B) Structure of phenylpropanoids. (C) Structure of lignans. (D) Structure of iridoids. (E) Structure of polyphenol. (F) Structure of saponins. (G) Structure of other type compounds. Figure S2: the cracking pathways of (A) ginsenoside Rb1, (B) notoginsenoside R1, and (C) 3-(β-D-glucopyranosyl-β-D-glucopyranosyl)-20-O-(6-O-malonyl-β-D-glucopyranosyl-β-D-glucopyranosyl)-3β,12β, 20(S)-trihydroxydammar-24-ene. Figure S3: the cracking pathways of (A) rutin, (B) chlorogenic acids, (C) deacetyl asperulosidic acid. Figure S4: the content of eight batches of medicinal materials. [file 9255305.f1.doc]

**Table S1** The final concentration of 19 standard solution.

| **Compound** | **The first point** | **The second point** | **The third point** | **The fourth point** | **The fifth point** | **The sixth point** |
| --- | --- | --- | --- | --- | --- | --- |
|  | **μg/mL** | **μg/mL** | **μg/mL** | **μg/mL** | **μg/mL** | **μg/mL** |
| ICGAA | 4.800 | 9.600 | 24.000 | 48.000 | 96.000 | 192.000 |
| 1,5-DQA | 6.100 | 12.200 | 30.500 | 61.000 | 122.000 | 244.000 |
| GE | 0.023 | 0.046 | 0.115 | 0.230 | 0.460 | 0.920 |
| APG | 0.065 | 0.130 | 0.325 | 0.650 | 1.300 | 2.600 |
| LT | 0.015 | 0.030 | 0.075 | 0.150 | 0.300 | 0.600 |
| KPF | 0.010 | 0.020 | 0.050 | 0.100 | 0.200 | 0.400 |
| QC | 0.011 | 0.022 | 0.055 | 0.110 | 0.220 | 0.440 |
| A-7-O-G | 4.200 | 8.400 | 21.000 | 42.000 | 84.000 | 168.000 |
| RU | 0.230 | 0.460 | 1.150 | 2.300 | 4.600 | 9.200 |
| HSYA | 9.830 | 19.660 | 49.150 | 98.300 | 196.600 | 393.200 |
| NG-R1 | 2.400 | 4.800 | 12.000 | 24.000 | 48.000 | 96.000 |
| G-Re | 1.010 | 2.020 | 5.050 | 10.100 | 20.200 | 40.400 |
| G-Rg1 | 6.500 | 13.000 | 32.500 | 65.000 | 130.000 | 260.000 |
| G-Rb1 | 5.660 | 11.320 | 28.300 | 56.600 | 113.200 | 226.400 |
| CA | 0.260 | 0.520 | 1.300 | 2.600 | 5.200 | 10.400 |
| FA | 0.390 | 0.780 | 1.950 | 3.900 | 7.800 | 15.600 |
| GPA | 0.800 | 1.600 | 4.000 | 8.000 | 16.000 | 32.000 |
| CGA | 5.500 | 11.000 | 27.500 | 55.000 | 110.000 | 220.000 |
| PDG | 2.180 | 4.360 | 10.900 | 21.800 | 43.600 | 87.200 |

**Table S2** The content of 19 compounds in the MTBD medicinal measured (μg, `X ± SD, n=3).

| **Compound** | **Batch 1** | | **Batch 2** | | **Batch 3** | | **Batch 4** | | **Batch 5** | | **Batch 6** | | **Batch 7** | | **Batch 8** | |
| --- | --- | --- | --- | --- | --- | --- | --- | --- | --- | --- | --- | --- | --- | --- | --- | --- |
|  | **Content**  **(μg)** | ***RSD***  **(%)** | **Content**  **(μg)** | ***RSD***  **(%)** | **Content**  **(μg)** | ***RSD***  **(%)** | **Content**  **(μg)** | ***RSD***  **(%)** | **Content**  **(μg)** | ***RSD***  **(%)** | **Content**  **(μg)** | ***RSD***  **(%)** | **Content**  **(μg)** | ***RSD***  **(%)** | **Content**  **(μg)** | ***RSD***  **(%)** |
| ICGAA | 16762.18 ±  199.87 | 1.19 | 11295.32±  149.83 | 1.33 | 13711.97 ±  574.45 | 4.19 | 14877.31 ±  411.70 | 2.77 | 18213.17 ±  604.40 | 3.32 | 17164.28±  553.79 | 3.23 | 16781.86±  743.30 | 2.92 | 16781.87±  743.30 | 4.43 |
| 1,5-DQA | 13869.07 ±  653.18 | 4.71 | 12125.54±  324.70 | 2.68 | 3151.23 ±  42.20 | 1.34 | 9869.47±  186.95 | 1.89 | 14198.79 ±  468.05 | 3.30 | 15605.54±  768.51 | 4.92 | 5106.09±  189.07 | 3.70 | 11689.73±  478.39 | 4.09 |
| GE | 21.46 ±  1.26 | 4.73 | 12.26 ±  0.03 | 0.21 | 7.16 ±  0.17 | 2.35 | 29.67 ±  0.49 | 1.67 | 24.35 ±  1.46 | 6.03 | 18.19 ±  0.41 | 2.27 | 11.35 ±  0.46 | 4.11 | 35.57 ±  1.25 | 3.54 |
| APG | 193.44 ±  7.91 | 4.09 | 30.00 ±  0.51 | 1.73 | 180.55 ±  7.09 | 3.93 | 61.60 ±  2.16 | 3.52 | 173.06 ±  9.52 | 5.50 | 26.22 ±  0.97 | 3.73 | 221.23 ±  5.14 | 2.33 | 70.88 ±  3.77 | 5.32 |
| LT | 27.43 ±  1.31 | 4.80 | 9.46 ±  0.46 | 4.87 | 28.72 ±  1.06 | 3.70 | 11..38 ±  0.49 | 4.32 | 24.46 ±  0.85 | 3.49 | 6.33 ±  0.36 | 5.74 | 38.02 ±  1.77 | 4.66 | 9.97 ±  0.39 | 3.98 |
| KPF | 8.76 ±  0.18 | 2.13 | 5.67 ±  0.10 | 1.90 | 7.52 ±  0.22 | 2.96 | 5.84 ±  0.27 | 4.76 | 11.25 ±  0.28 | 2.46 | 7.14 ±  0.24 | 3.31 | 15.15 ±  0.86 | 5.67 | 8.03 ±  0.47 | 5.83 |
| QC | 5.88 ±  0.04 | 0.64 | 3.89 ±  0.06 | 1.46 | 5.65 ±  0.29 | 5.15 | 4.32 ±  0.16 | 3.77 | 8.00 ±  0.32 | 4.03 | 4.29 ±  0.21 | 4.79 | 9.29 ±  0.26 | 2.81 | 5.37 ±  0.23 | 4.24 |
| A-7-O-G | 1338.14 ±  56.00 | 4.18 | 522.55 ±  12.41 | 2.37 | 2642.11 ±  111.36 | 4.21 | 1005.98 ±  28.87 | 2.87 | 1485.53 ±  73.13 | 4.92 | 418.74 ±  15.29 | 3.65 | 3759.35 ±  132.56 | 3.53 | 1132.32 ±  56.29 | 4.97 |
| RU | 278.13 ±  11.11 | 4.00 | 249.04 ±  5.18 | 2.08 | 224.49 ±  5.36 | 2.39 | 272.52 ±  8.27 | 3.03 | 299.01 ±  9.27 | 3.10 | 405.94 ±  10.02 | 2.47 | 353.85 ±  6.71 | 1.90 | 811.46 ±  19.71 | 2.43 |
| HSYA | 36875.52 ±  584.45 | 1.58 | 32683.88±  1097.05 | 3.36 | 32605.85 ±  1720.72 | 5.28 | 36899.09 ±  1252.44 | 3.39 | 36566.99 ±  1173.24 | 3.21 | 37101.52±  865.43 | 2.33 | 35625.68±  1346.05 | 3.78 | 37826.12±  1318.17 | 3.48 |
| NG-R1 | 2286.91 ±  110.69 | 4.84 | 1761.26 ±  79.66 | 4.52 | 2534.44 ±  68.48 | 2.70 | 2245.17 ±  104.11 | 4.64 | 3040.46 ±  147.89 | 4.86 | 2719.07 ±  86.30 | 3.17 | 3063.47 ±  91.59 | 2.99 | 2848.93 ±  175.01 | 6.14 |
| G-Re | 115.34 ±  2.04 | 1.18 | 173.72 ±  2.04 | 1.18 | 138.15 ±  3.66 | 2.65 | 128.01 ±  2.95 | 2.30 | 129.25 ±  3.70 | 2.86 | 154.83 ±  4.72 | 3.05 | 117.29 ±  1.20 | 1.02 | 107.36 ±  3.29 | 3.07 |
| G-Rg1 | 16762.12 ±  199.87 | 1.19 | 21295.33±  149.83 | 1.33 | 13711.97 ±  574.45 | 4.19 | 14877.31 ±  411.70 | 2.77 | 18213.17 ±  604.40 | 3.32 | 17164.28±  553.80 | 3.23 | 18534.92±  541.17 | 2.92 | 16781.87±  743.30 | 4.43 |
| G-Rb1 | 8989.89 ±  161.19 | 1.79 | 8170.53 ±  276.59 | 3.39 | 8514.05 ±  329.66 | 3.87 | 8002.95 ±  114.29 | 1.43 | 10322.41 ±  184.74 | 1.79 | 11080.66±  554.08 | 5.00 | 9488.45 ±  406.75 | 4.29 | 9351.60 ±  137.92 | 1.47 |
| CA | 684.33 ±  0.99 | 0.14 | 555.93 ±  19.10 | 3.44 | 980.54±  29.49 | 3.01 | 362.15 ±  16.41 | 4.53 | 601.07 ±  11.68 | 1.94 | 489.06 ±  13.84 | 2.83 | 953.46 ±  25.03 | 2.63 | 394.46 ±  14.01 | 3.55 |
| FA | 142.20 ±  2.60 | 1.83 | 208.53 ±  6.40 | 3.07 | 211.98 ±  7.80 | 3.68 | 211.14 ±  9.22 | 4.17 | 271.42 ±  1.19 | 0.44 | 468.62 ±  8.42 | 1.80 | 532.06 ±  12.35 | 2.32 | 558.66 ±  15.01 | 2.69 |
| GPA | 3146.68 ±  108.83 | 3.46 | 3163.53 ±  123.27 | 3.90 | 3112.44 ±  116.01 | 3.73 | 3045.59 ±  86.91 | 2.85 | 3163.44 ±  41.17 | 1.30 | 3284.01 ±  73.66 | 2.24 | 3173.78 ±  118.37 | 3.73 | 3355.00 ±  98.55 | 2.94 |
| CGA | 11069.83 ±  373.70 | 3.38 | 21193.91±  16.47 | 0.08 | 6736.66 ±  230.72 | 3.42 | 11708.75 ±  230.42 | 1.97 | 11213.11 ±  322.76 | 2.88 | 20884.47±  710.15 | 3.40 | 8027.72 ±  378.50 | 4.71 | 12505.65±  186.75 | 1.49 |
| PDG | 8372.22 ±  65.92 | 0.79 | 7228.23 ±  224.48 | 3.11 | 8303.93 ±  75.14 | 0.90 | 8145.20 ±  244.72 | 3.00 | 7812.40 ±  325.53 | 4.17 | 6784.62 ±  257.26 | 3.79 | 8551.33 ±  250.91 | 2.93 | 8325.58 ±  77.24 | 0.93 |

**Table S3** Different sources of medicinal materials

| **NO** | **EU** | **ELT** | **CT** | **PN** |
| --- | --- | --- | --- | --- |
| **Batch 1** | purchased from Bozhou | collected from Hohhot | purchased from Guoda drug store | purchased from Bozhou |
| **Batch 2** | purchased from Bozhou | collected from Ordos | purchased from Guoda drug store | purchased from Bozhou |
| **Batch 3** | purchased from Bozhou | collected from Xilingol | purchased from Guoda drug stor | purchased from Bozhou |
| **Batch 4** | purchased from Bozhou | collected from Ulan Hot | purchased from Guoda drug stor | purchased from Bozhou |
| **Batch 5** | purchased from Guoda drug store | collected from Hohhot | purchased from Bozhou | purchased from Guoda drug store |
| **Batch 6** | purchased from Guoda drug store | collected from Ordos | purchased from Bozhou | purchased from Guoda drug store |
| **Batch 7** | purchased from Guoda drug store | collected from Xilingol | purchased from Bozhou | purchased from Guoda drug storeu |
| **Batch 8** | purchased from Guoda drug store | collected from Ulan Hot | purchased from Bozhou | purchased from Guoda drug store |

**Table S4** Quality control concentration levels of 19 compounds.

| **Compound** | **Low level**  **(μg/mL)** | **Medium leve**  **(μg/mL)l** | **High level**  **(μg/mL)** |
| --- | --- | --- | --- |
| ICGAA | 6.000 | 60.000 | 153.600 |
| 1,5-DQA | 7.625 | 76.250 | 195.200 |
| GE | 0.029 | 0.288 | 0.736 |
| APG | 0.081 | 0.813 | 2.080 |
| LT | 0.019 | 0.188 | 0.480 |
| KPF | 0.013 | 0.125 | 0.320 |
| QC | 0.014 | 0.138 | 0.352 |
| A-7-O-G | 5.250 | 52.500 | 134.400 |
| RU | 0.288 | 2.875 | 7.360 |
| HSYA | 12.290 | 123.000 | 310.000 |
| NG-R1 | 3.000 | 30.000 | 76.800 |
| G-Re | 1.263 | 12.625 | 32.320 |
| G-Rg1 | 8.125 | 81.250 | 208.000 |
| G-Rb1 | 7.075 | 70.750 | 181.120 |
| CA | 0.325 | 3.250 | 8.320 |
| FA | 0.488 | 4.875 | 12.480 |
| GPA | 1.000 | 10.000 | 30.000 |
| CGA | 6.875 | 68.750 | 176.000 |
| PDG | 2.730 | 27.000 | 70.000 |


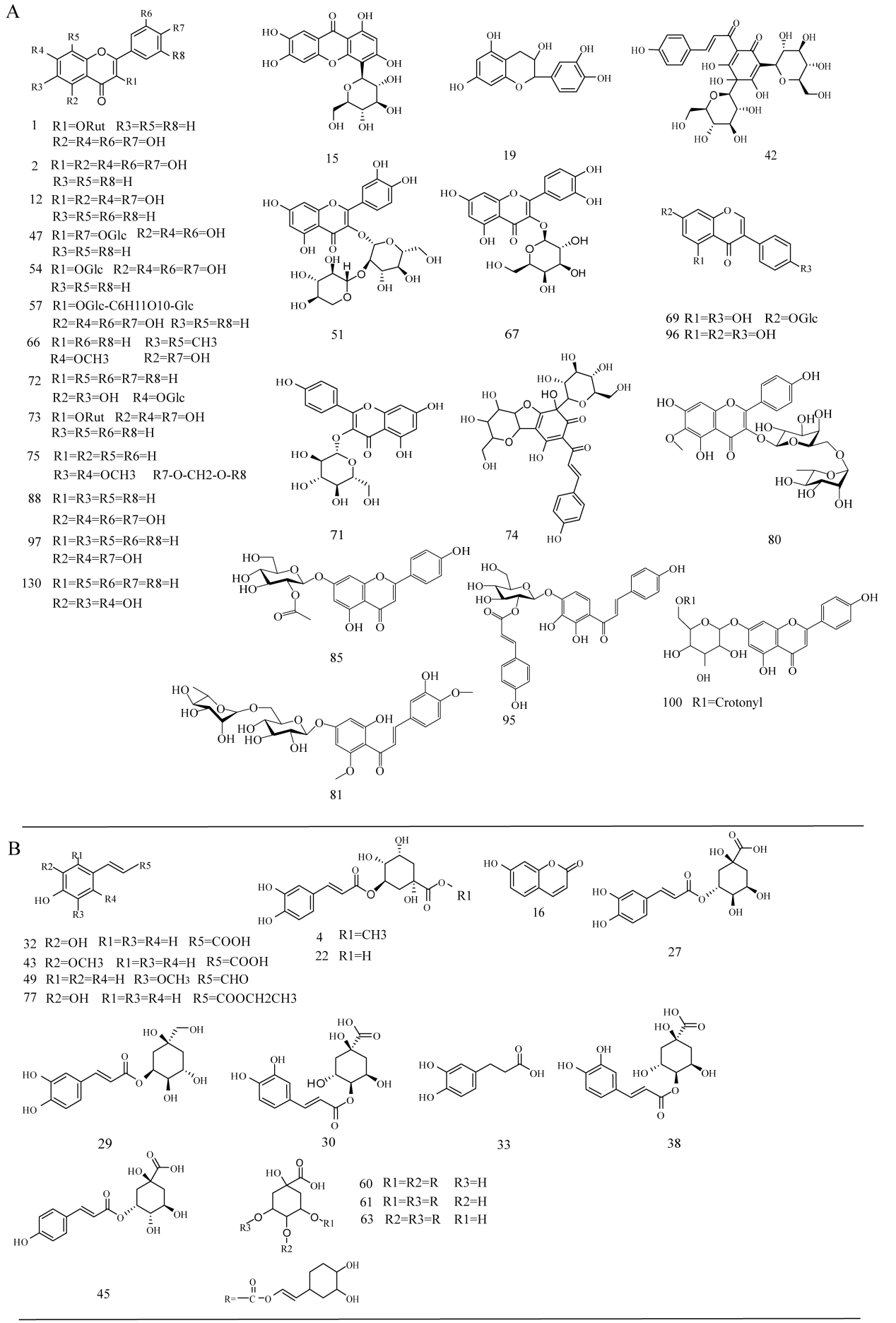


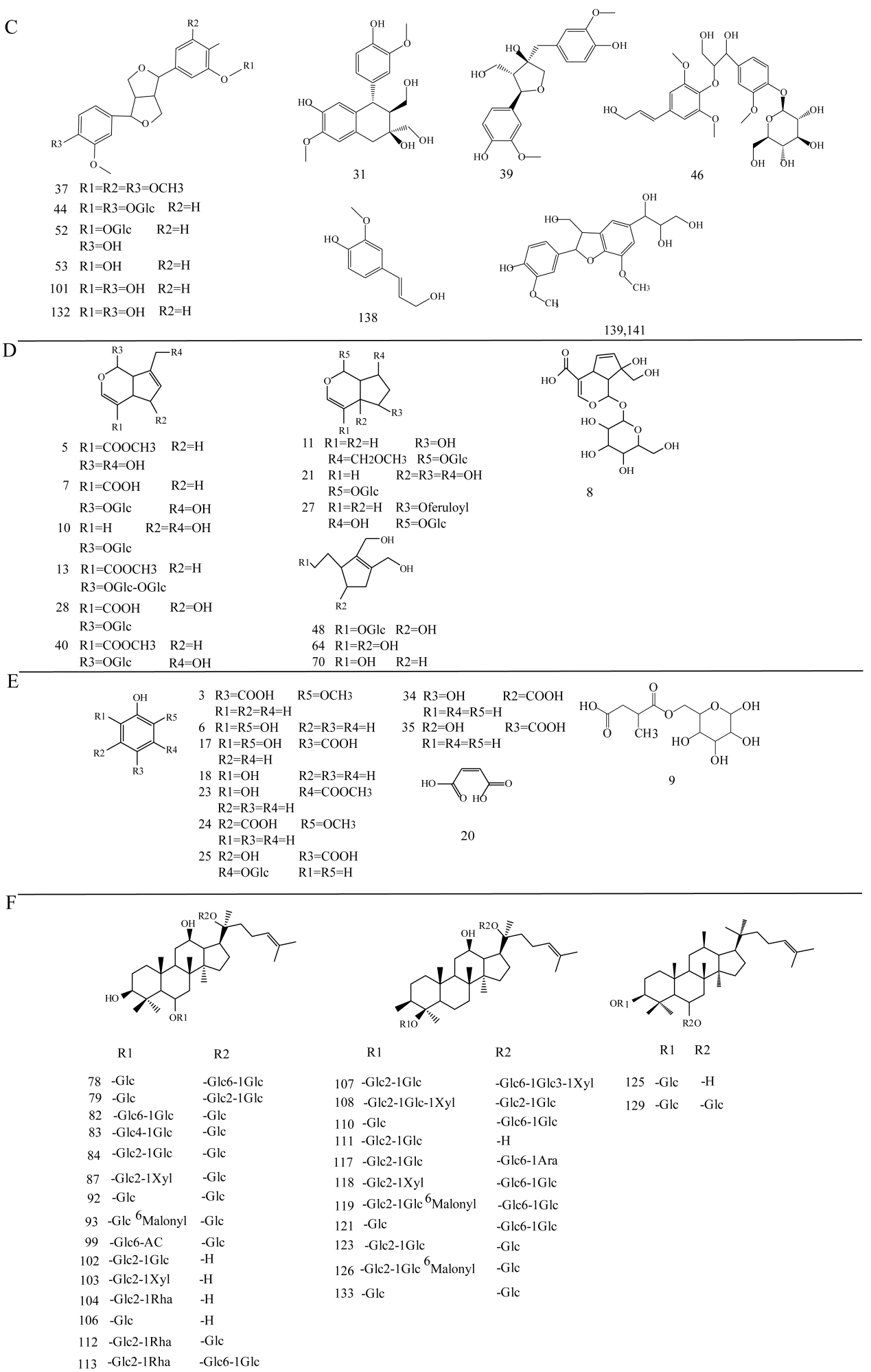


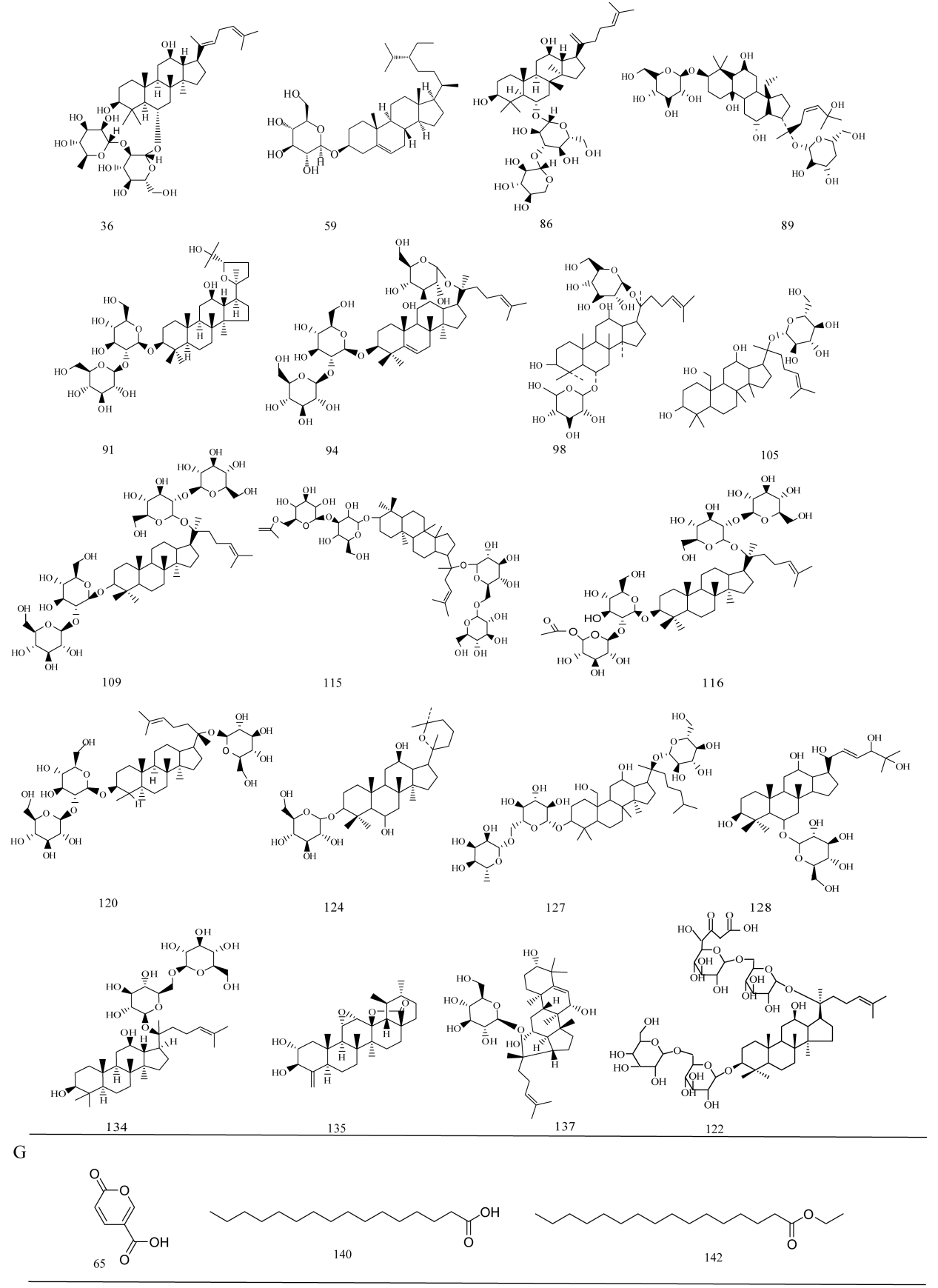


**Figure S1**. Structures of 143 compounds. (A) Structure of flavonoids (B) Structure of phenylpropanines (C) Structure of lignans (D) Structure of irilldoids (E) Structure of polyphenol (F) Structure of saponins (G) Structure of other type compounds


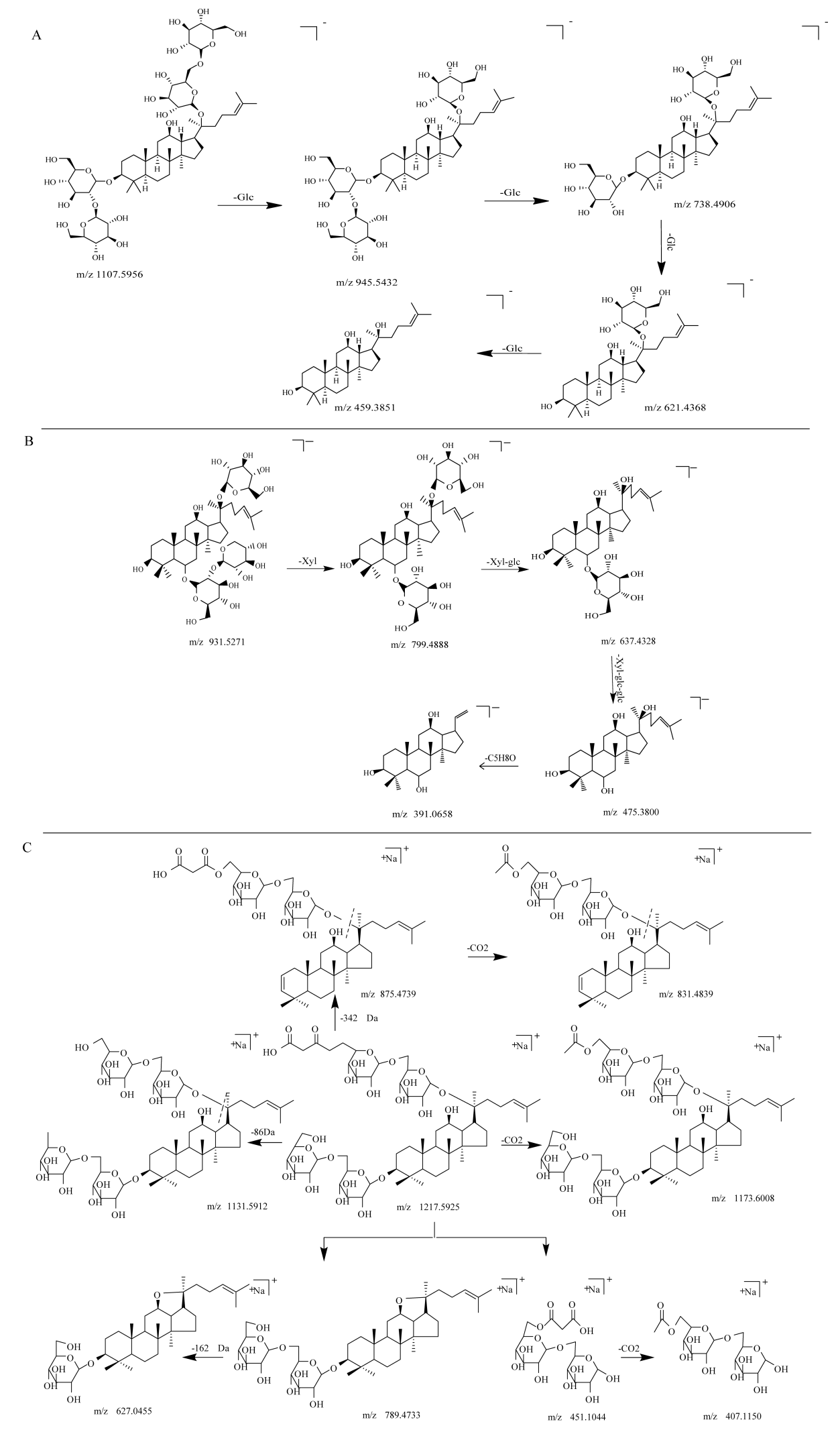


**Figure S2**. The cracking pathways of (A) ginsenoside Rb_1_, (B) notoginsenoside R_1_, (C) 3-(β-D-glucopyranosyl-β-D-glucopyranosyl)-20-O-(6-O-malonyl-β-D-glucopyranosyl-β-D-glucopyranosyl)-3β,12β,20(S)-trihydroxydammar-24-ene.


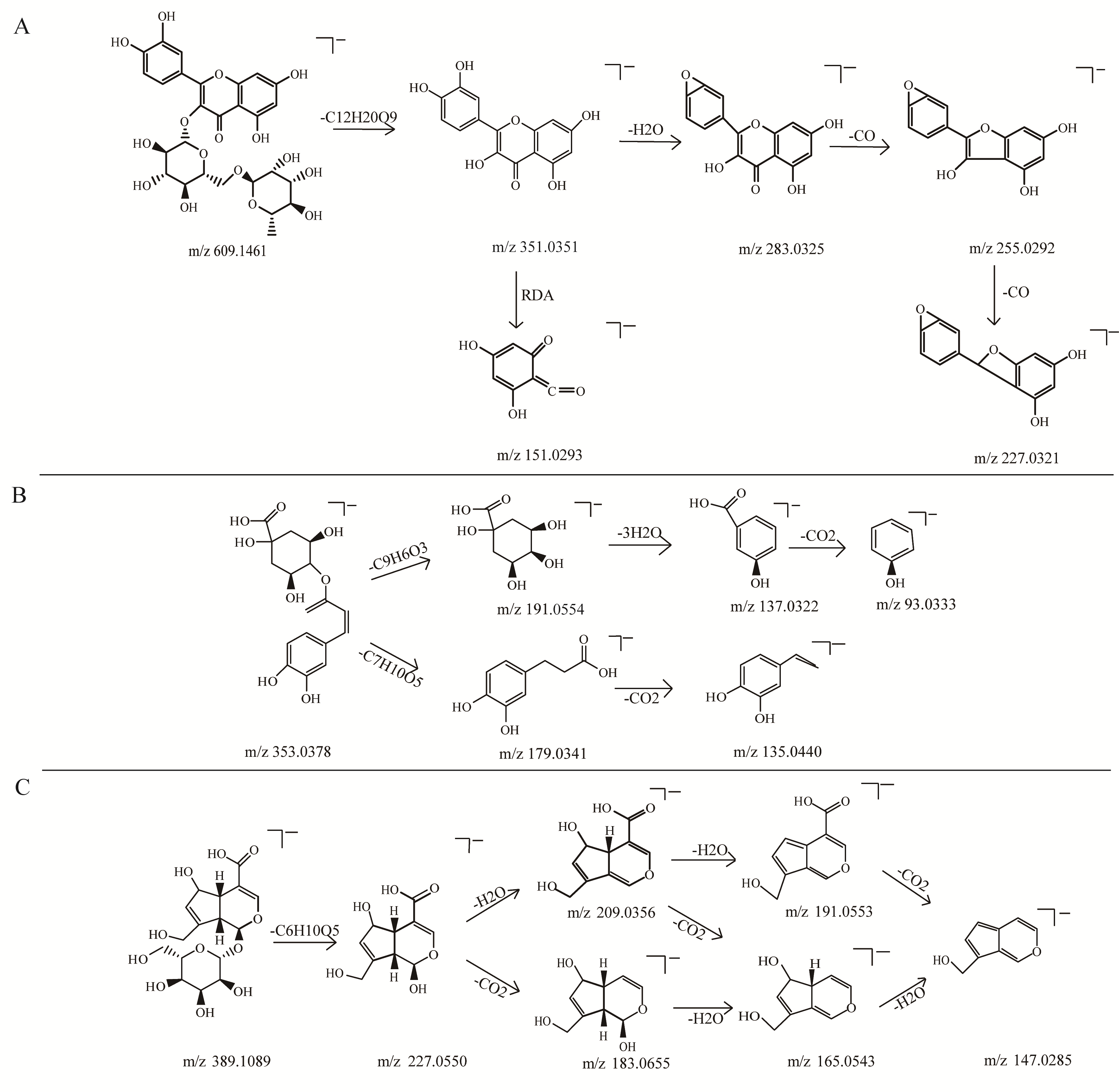


**Figure S3**. The cracking pathways of (A) rutin, (B) chlorogenic acids, (C) deacetyl trifolate.


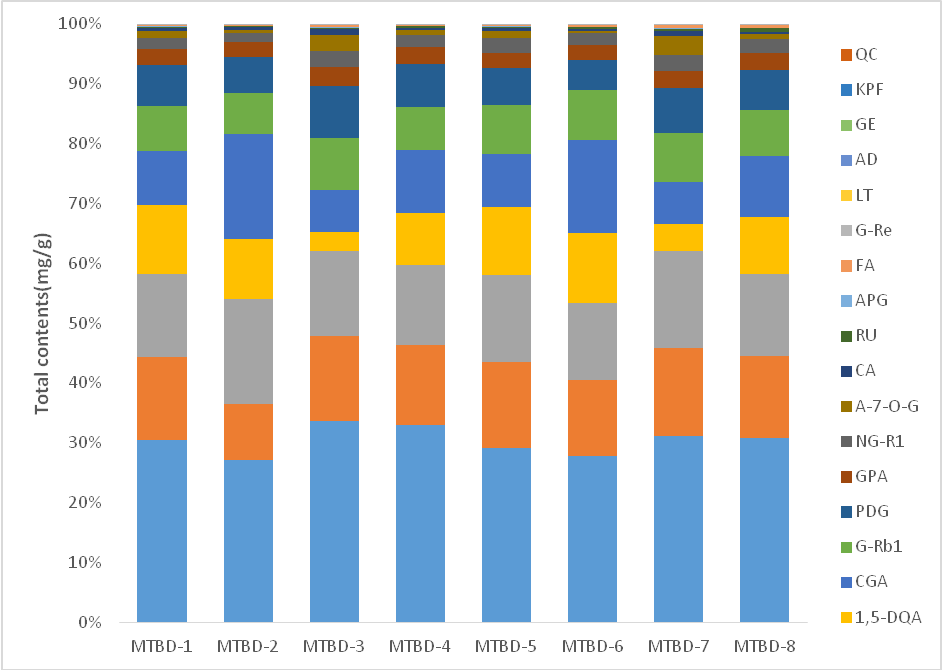


**Figure S4.** The content of eight batches of medicinal materials.
